# Invasion of epithelial cells by *Campylobacter jejuni* is independent of caveolae

**DOI:** 10.1186/1478-811X-11-100

**Published:** 2013-12-23

**Authors:** Michael E Konkel, Derrick R Samuelson, Tyson P Eucker, Eric A Shelden, Jason L O'Loughlin

**Affiliations:** 1 School of Molecular Biosciences, College of Veterinary Medicine, Washington State University, Life Sciences Bldg. Room 302c, Pullman, WA, USA

**Keywords:** Lipid rafts, Caveolin-1, Cell signaling, Focal complex, Membrane ruffling

## Abstract

Caveolae are 25–100 nm flask-like membrane structures enriched in cholesterol and glycosphingolipids. Researchers have proposed that *Campylobacter jejuni* require caveolae for cell invasion based on the finding that treatment of cells with the cholesterol-depleting compounds filipin III or methyl-β-cyclodextrin (MβCD) block bacterial internalization in a dose-dependent manner. The purpose of this study was to determine the role of caveolae and caveolin-1, a principal component of caveolae, in *C. jejuni* internalization. Consistent with previous work, we found that the treatment of HeLa cells with MβCD inhibited *C. jejuni* internalization. However, we also found that the treatment of HeLa cells with caveolin-1 siRNA, which resulted in greater than a 90% knockdown in caveolin-1 protein levels, had no effect on *C. jejuni* internalization. Based on this observation we performed a series of experiments that demonstrate that MβCD acts broadly, disrupting host cell lipid rafts and *C. jejuni-*induced cell signaling. More specifically, we found that MβCD inhibits the cellular events necessary for *C. jejuni* internalization, including membrane ruffling and Rac1 GTPase activation. We also demonstrate that MβCD disrupted the association of the β_1_ integrin and EGF receptor, which are required for the maximal invasion of epithelial cells. In agreement with these findings, *C. jejuni* were able to invade human Caco-2 cells, which are devoid of caveolae, at a level equal to that of HeLa cells. Taken together, the results of our study demonstrate that *C. jejuni* internalization occurs in a caveolae-independent manner.

## Lay abstract

*Campylobacter jejuni* is responsible for a significant proportion of human morbidity and mortality in both developing and developed countries. Most cases of campylobacteriosis result from consumption of foods cross-contaminated with undercooked chicken products. Acute disease is dependent upon the ability of *C. jejuni* to bind and invade the cells lining the human gastrointestinal tract. While significant progress has been made in identifying and characterizing the bacterial components that contribute to the development of disease in humans, how the bacterium manipulates the host intestinal cells during infection is less well-defined. For more than a decade researchers have proposed that *C. jejuni* invasion of intestinal cells requires specialized structures called caveolae. We present evidence demonstrating that *C. jejuni* internalization is not dependent on caveolae, but requires the cellular components that comprise the focal complex. Our data provides new insight into the mechanism that *C. jejuni* utilizes to invade intestinal cells. Elucidation of the mechanism of *C. jejuni* cell invasion will aid in the development of novel intervention methods to reduce human disease.

## Background

*Campylobacter jejuni* is one of the leading bacterial causes of human gastrointestinal disease worldwide. Clinical and experimental research demonstrates that acute disease involves *C. jejuni* invasion of the cells lining the gastrointestinal tract [1-5]. While progress has been made in identifying *C. jejuni* virulence determinants, the mechanism of cell invasion and the host cell components involved in *C. jejuni* uptake are less well-defined.

Lipid rafts are distinct regions of the plasma membrane that contain high concentrations of cholesterol and glycosphingolipids [6]. Caveolae are a special type of lipid raft. Caveolar membranes contain caveolins, which bind cholesterol and form complexes with glycosphingolipids (GSLs) and glycosyl phosphatidyl inositol (GPI) anchored proteins [7]. Three members of the caveolin gene family have been identified (caveolin-1, caveolin-2, and caveolin-3). Caveolin-1, a 21 to 24-kDa integral membrane protein, is a principal component of caveolar membranes and a major component of the vesicular transport system in the *trans*-Golgi network [8,9]. Caveolin-2 tightly interacts with caveolin-1. More specifically, the interaction with caveolin-1 is necessary for transport of caveolin-2 to the plasma membrane, where the two proteins form hetero-oligomeric complexes within caveolae [10]. Caveolin-2 is a minor component of the hetero-oligomeric complexes, and is readily degraded in the absence of caveolin-1 [11]. Caveolin-2 has been proposed to act as a co-factor for caveolae formation, regulating the size and shape of the structures. Relevant to this study, caveolin-2 is not necessary for caveolae formation, and caveolin-1 and caveolin-2 are not expressed in all cells [10,12]. In contrast to caveolin-1 and caveolin-2, caveolin-3 is only expressed in striated muscle [13,14].

Evidence from a number of *in vitro* studies has suggested that caveolae play a role in *C. jejuni* invasion. Wooldridge *et al*. [15] demonstrated that treatment of Caco-2 cells with the polyene antifungal agent filipin III, which binds to and sequesters cholesterol in the membrane [8,16], inhibited *C. jejuni* internalization of human Caco-2 cells in a dose-dependent manner [15]. A decade later Hu *et al*. [17] performed similar experiments using human INT 407 epithelial cells, and found that treatment of these cells with filipin III resulted in a dose-dependent reduction in *C. jejuni* invasion [17]. Similarly, Watson and Galan found that the treatment of human T84 cells with the cholesterol-depleting compound methyl-β-cyclodextrin (MβCD) blocked *C. jejuni* internalization in a dose-dependent manner [45]. These investigators also reported that transfection of Cos-1 fibroblast-like cells with a dominant-negative (DN) mutant of caveolin-1 (caveolin-1 Tyr-14 F), which prevents caveolin-1 activation by preventing the phosphorylation of tyrosine-14, significantly decreased *C. jejuni* internalization. To further dissect the importance of caveolae in *C. jejuni* internalization, the Cos-1 cells were transfected with a dominant-negative form of dynamin II (dynII K44A) to inhibit caveolae-dependent endocytosis. In contrast to the cells expressing the DN form of caveolin-1, *C. jejuni* internalization was not inhibited in cells expressing the DN form of dynamin II. The investigators concluded that the role of caveolin-1 in *C. jejuni* internalization might not be related to its role in caveolae-mediated endocytosis, but that caveolae or caveolin-1 may play a role in the host cell signaling events necessary for bacterial uptake. As recent as 2012, investigators proposed a model of *C. jejuni* internalization involving caveolae structures [18].

We have previously proposed a model of *C. jejuni-*mediated invasion whereby this pathogen activates numerous components that comprise the focal complex, resulting in cytoskeletal rearrangement and bacterial internalization [19]. Focal complexes are dynamic cellular structures that form transient attachments, often at the tip of a cellular protrusion. They connect extracellular matrix (ECM) components, including fibronectin, to the actin cytoskeleton and anchor the cell to the underlying surface. Focal complexes are comprised of integrin receptors, adaptor proteins (e.g., paxillin), signaling proteins (e.g., focal adhesion kinase (FAK)), and actin. We have found that the binding of *C. jejuni* to fibronectin induces the phosphorylation of paxillin, indicating host cell signal transduction from the extracellular matrix through the α_5_β_1_ integrin receptors [20]. *C. jejuni* internalization is dependent upon the activation of paxillin, Src, FAK, and Dock180 at the sites of bacterial invasion [19]. Finally, *C. jejuni* is responsible for the activation of the Rho GTPases Cdc42 and Rac1, which induce the host cell membrane ruffling necessary for bacterial uptake [19,21]. Interestingly, inhibitors that prevent the activation of the Epidermal Growth Factor (EGF) receptor also inhibit *C. jejuni* internalization [19]. In summary, *C. jejuni* can activate components of the focal complex, which in turn interact with other host cell scaffold and signaling proteins including the EGF receptor.

The purpose of this study was to determine the role of caveolae and caveolin-1, the principal marker protein of caveolae, in *C. jejuni* internalization. We demonstrate that caveolin-1 is associated with the active form of the EGF receptor in response to *C. jejuni* infection, but that caveolin-1 is not required for *C. jejuni* internalization. The results of our studies support the proposal that *C. jejuni* internalization is dependent upon activation of components of the focal complex.

## Results

### Part I. *C. jejuni* cell invasion, but not Cia protein delivery, is inhibited by MβCD treatment

#### C. jejuni *invasion is sensitive to treatment of cells with MβCD*

**Figure 1:**
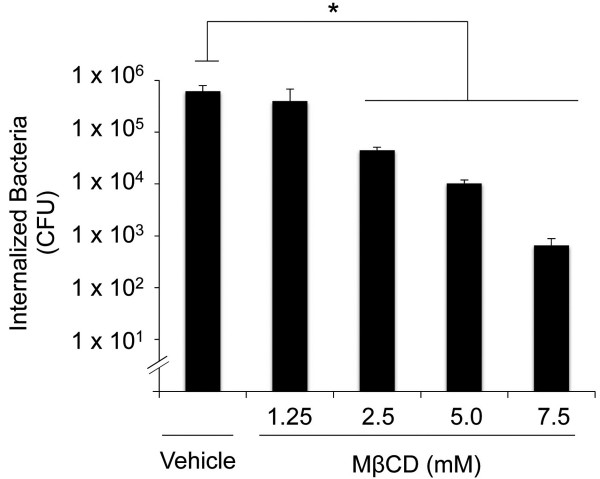
**Treatment of HeLa cells with the cholesterol-depleting compound methyl-β-cyclo-dextrin (MβCD) reduces *C. jejuni *internalization.** HeLa cells were treated with 1.25, 2.5, 5.0, and 7.5 mM of MβCD for 30 min prior to inoculation with *C. jejuni*, as outlined in the ‘Methods’ section. The control consisted of cells infected with *C. jejuni* in the absence of the inhibitor in medium containing vehicle (water). Bars indicate the mean of the number of internalized bacteria. The asterisks indicate a significant reduction in *C. jejuni* internalization compared to cells infected with *C. jejuni* in the absence of the inhibitor in medium alone, as judged by one-way ANOVA followed by post-hoc Dunnet’s analysis (*P* < 0.05). Each error bar represents ± the standard deviation of the mean (SD).

Researchers have concluded that the uptake of *C. jejuni* by host cells is dependent upon caveolae based on the finding that the treatment of epithelial cells with cholesterol-sequestering and cholesterol-depleting compounds, including filipin III and MβCD, inhibit *C. jejuni* invasion [15,17]. Consistent with previous reports, we found that treatment of HeLa cells with MβCD reduced *C. jejuni* internalization in a dose-dependent manner (Figure 1). Noteworthy is that treatment of HeLa cells with MβCD had no effect on *C. jejuni* binding to the epithelial cells (not shown) and importantly, the level of *C. jejuni* invasion was restored to that of untreated cells when the cells pre-treated with MβCD were supplemented with cholesterol prior to the infection (Additional file 1: Figure S1). Unsurprisingly, the cellular localization of caveolin-1 in HeLa cells treated with MβCD was different from untreated cells as judged by immunofluorescence microscopy (Additional file 2: Figure S2). To ensure that the effect of MβCD on *C. jejuni* internalization was not unique to this chemical compound*,* similar experiments were performed with 2-hydroxypropyl-β-cyclodextrin (HPβCD). Treatment of HeLa cells with HPβCD, which also promotes extensive release of cholesterol from cells, reduced *C. jejuni* internalization in a dose-dependent manner (Additional file 3: Figure S3). A greater reduction was observed in the number of *C. jejuni* internalized in MβCD treated cells versus HPβCD treated cells, which is consistent with the previous findings that indicate that MβCD is more potent than HPβCD at extracting cholesterol from biological membranes [22]. Treatment of cells with filipin III or nystatin, which are cholesterol-sequestering agents [8,16,23], led to a moderate increase in *C. jejuni* internalization (Additional file 4: Figure S4). This result is consistent with recent findings with *Francisella*[24]. The fact that *C. jejuni* internalization is inhibited by MβCD and HPβCD is consistent with the hypothesis that efficient cell invasion requires the presence of cholesterol in the plasma membrane.

#### C. jejuni *membrane ruffling is sensitive to treatment of cells with MβCD*

**Figure 2:**
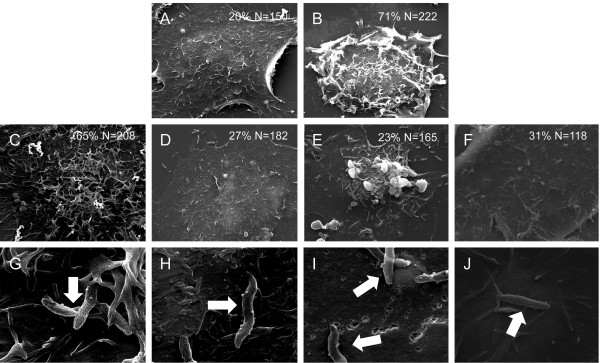
***C. jejuni*-induced membrane ruffling is dependent on functional lipid rafts, microtubules, and microfilaments, as judged by scanning electron microscopy (SEM) examination of cells.** Negative and positive controls consisted of uninfected (Panel **A**) and EGF-treated HeLa cells (Panel **B**). SEM images of HeLa cells infected with *C. jejuni* without an inhibitor (Panels **C** and **G**) and in the presence of methyl-β-cyclodextrin (MβCD) (Panels **D** and **H**), cytochalasin D (Panels **E** and **I**), and nocodazole (Panels **F** and **J**). Indicated within the figure is the percentage of cells with membrane ruffling of N number of total cells. Arrows indicate *C. jejuni* associated with host cells. The images shown in Panels **A-F** were collected at the same magnification and the images shown in Panels **G-J** were collected at the same magnification.

Assays were performed to determine if *C. jejuni* were able to induce membrane ruffling in HeLa cells treated with MβCD, as previous studies have indicated that host cell membrane ruffling is required for *C. jejuni* cell invasion [19,21]. We chose to use MβCD rather than HPβCD for this experiment and in many of the other of the experiments performed in this study, as it was found to be a more potent inhibitor of *C. jejuni* internalization. We also treated the epithelial cells with nocodazole and cytochalasin D, in part as controls, as these inhibitors have been reported to reduce *C. jejuni* internalization. Nocodazole binds β-tubulin, thereby preventing tubulin polymerization [25], whereas cytochalasin D inhibits actin polymerization and transient integrin-stimulated focal adhesion kinase (FAK) activation [26]. The HeLa cells were pre-treated for 30 min with MβCD, nocodazole, and cytochalasin D to target host cell processes, inoculated with *C. jejuni*, and then examined by SEM for membrane ruffling. EGF treated cells served as the positive control, whereas uninoculated HeLa cells were used as a negative control. EGF treatment of epithelial cells was used as a positive control because it can rescue a *C. jejuni* invasion-deficient mutant, in part, because it stimulates membrane ruffling [19]. We observed that 65.0% (*n* = 135 of 208) of the cells infected with *C. jejuni* showed membrane ruffling (Figure 2). In contrast, membrane ruffling was significantly reduced (~38%, *P* < 0.05) in *C. jejuni*-infected cells that were pre-treated with MβCD, nocodazole, and cytochalasin D (Figure 2). Treatment of HeLa cells with MβCD, nocodazole, and cytochalasin D did not result in cell death as judged by trypan blue staining (not shown). These findings demonstrate that drugs that target different host cell structural components and processes can prevent *C. jejuni*-induced host cell membrane ruffling. These data further indicate that *C. jejuni* invasion of host cells is dependent upon lipid rafts, as a significant reduction in internalization was observed in epithelial cells with MβCD.

#### Treatment of HeLa cells with MβCD prevents Rac1 activation but not Cia protein delivery

**Figure 3:**
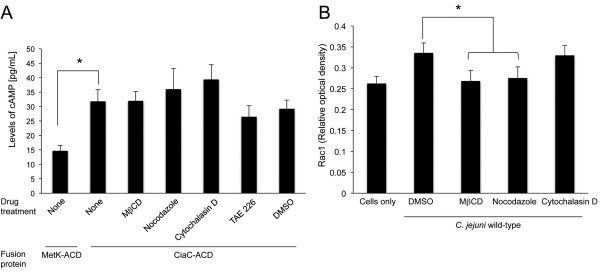
**Disruption of lipid rafts and microtubules, but not microfilaments, inhibits the activation of the Rho GTPase Rac1 in *C. jejuni*-infected HeLa cells.** Assays were performed to assess the delivery of the *C. jejuni* CiaC protein to the cytosol of HeLa cells (Panel **A**) and to determine the level of Rac1 activity in *C. jejuni*-infected HeLa cells (Panel **B**). HeLa cells were pre-treated methyl-β-cyclodextrin (MβCD) (5 mM), nocodazole (20 mM), cytochalasin D (1 mM), and TAE 226 (5 μM) and infected with *C. jejuni* as outlined in ‘the ‘Methods’ section. Panels: **A)** Delivery of the CiaC-ACD fusion protein from *C. jejuni* to the cytosol of HeLa cells was assayed using the adenylate cyclase domain (ACD) reporter delivery assay. The concentration of cAMP within HeLa cells was assayed 30 min post-infection. The error bars represent mean ± standard deviation (SD) from five independent experiments. The level of cAMP for cells infected with *C. jejuni* in the absence of an inhibitor was significantly greater than for the negative control MetK-ACD, as judged by one-way ANOVA followed by post-hoc Tukey’s analysis. None of the treatments resulted in a value significantly (*P* < 0.05) different from the control (HeLa cells infected with *C. jejuni* in the absence of a host cell inhibitor). **B)** Rac1 activation in host cells infected with *C. jejuni*. Whole cell lysates were analyzed for activated Rac1 by G-LISA™. The mean ± SD of total active Rac1 is indicated in Relative Optical Density. The data shown represent analysis of four samples. The asterisks indicate a significant difference (*P* < 0.01) in the level of Rac1 activation in cells infected with *C. jejuni* in the presence versus absence of a host cell inhibitor, as judged by one-way ANOVA followed by post-hoc Tukey’s analysis.

Maximal *C. jejuni* invasion of host cells requires the *Campylobacter* invasion antigens (Cia). The Cia proteins are synthesized and exported from the flagellar Type III Secretion System (T3SS) in response to the conditions that the bacterium encounters *in vivo*. We have found that CiaC is delivered to host cells and is necessary for the recruitment and activation of the Rho GTPase Rac1 [19,27]. Here we used the adenylate cyclase domain (ACD) reporter assay to determine if CiaC was delivered to the cytosol of HeLa cells pre-treated with MβCD, nocodazole, cytochalasin D, and TAE 226. The inhibitor TAE 226 specifically suppresses ECM-dependent phosphorylation of FAK at Tyr-397 and Tyr-861 [28]. Infection with *C. jejuni* synthesizing the CiaC-ACD fusion protein resulted in a significant increase in cytosolic cAMP in cells treated with each inhibitor compared to HeLa cells infected with *C. jejuni* synthesizing the MetK-ACD fusion protein (Figure 3A). MetK-ACD was used as a negative control in this assay, as the *metK* gene encodes for *S*-adenosylmethionine synthetase that is localized in the bacterial cytoplasm [29]. Based on these results, none of the drugs that targeted the host cell processes altered the delivery of the Cia proteins from the bacteria to the host cells.

Noteworthy is that *C. jejuni* internalization is dependent upon the activation of the Rho GTPases Rac1 and Cdc42 [19,21]. Moreover, the activation of Rac1 has been reported to result in membrane ruffles [30,31]. We used a Rac1 G-LISA™ to determine the level of activated Rac1 in HeLa cells pre-treated with MβCD, nocodazole, and cytochalasin D and infected with *C. jejuni.* In contrast to untreated and cytochalasin D treated cells that were infected with *C. jejuni*, a significant decrease was observed in the level of activated Rac1 in *C. jejuni* infected HeLa cells treated with MβCD and nocodazole (Figure 3B). Collectively, these data indicate that MβCD and nocodazole act to prevent or block the host signal transduction events leading to the activation of Rac1.

### Part II. *C. jejuni* in the process of cell invasion are associated with FC components

#### C. jejuni *is associated with components of the focal complex*

Based on previous work suggesting that *C. jejuni* internalization is dependent upon the recruitment and activation of components of the focal complex, confocal microscopy experiments were performed to determine if *C. jejuni* are co-localized with components of the focal complex [32]. Paxillin and vinculin are two key components that comprise the focal complex [33]. The association of *C. jejuni* with the host cell proteins paxillin and vinculin was examined 45 min post-inoculation by confocal microscopy. Although only a small fraction of bacteria are in the process of internalization, examination of the *C. jejuni*-infected HeLa cells readily revealed sites of *C. jejuni* co-localized with paxillin and vinculin (Additional file 5: Figure S5 A and B and Additional file 6: Figure S6). More specifically, quantitative analysis of the *C. jejuni*-infected HeLa cells revealed that more than 40% of the cell-associated bacteria were co-localized with paxillin and vinculin. Experiments were then performed to determine if MβCD treatment of HeLa cells effected the co-localization of *C. jejuni* with focal complex components. The treatment of HeLa cells with MβCD appeared to increase the size of the focal adhesion and resulted in a decrease in the number of cell-associated bacteria that were co-localized with paxillin and vinculin (Additional file 5: Figure S5 C and D).

#### Caveolin-1 is associated with the components of the focal complex

**Figure 4:**
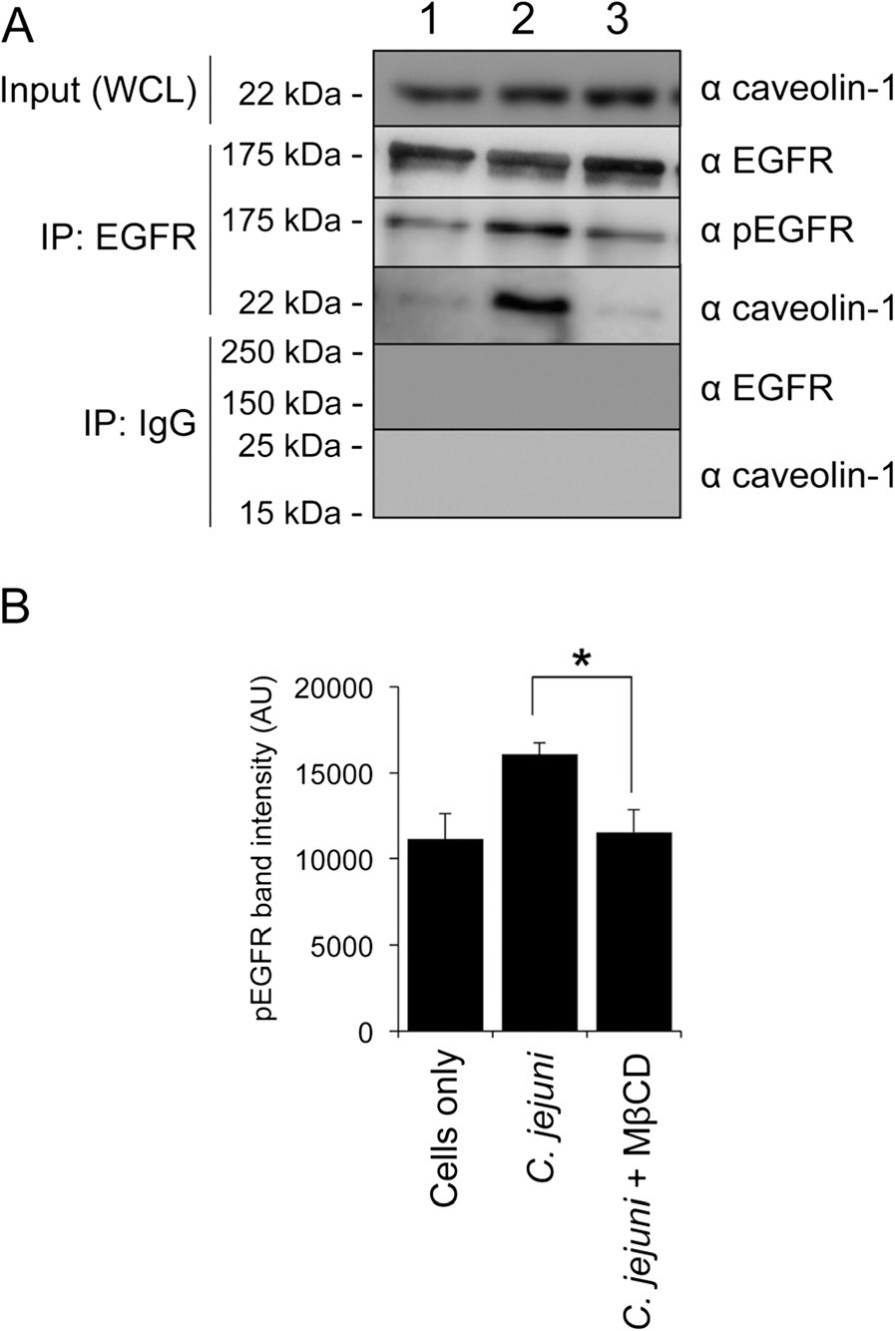
**Treatment of HeLa cells with MβCD disrupts *C. jejuni*-dependent EGF receptor (EGFR) phosphorylation and prevents EGFR association with caveolin-1.** HeLa cells were infected with *C. jejuni* with and without 5 mM MβCD treatment for 45 min. Panels: **A)** Cell lysates were immunoprecipitated (IP) with an EGFR antibody, separated by SDS-PAGE, and blotted for pEGFR, total EGFR, and caveolin-1. Lanes: 1, Uninfected cells (Cells only); 2, Infected with *C. jejuni* in the absence of MβCD (vehicle only, water); 3, Infected with *C. jejuni* in the presence of 5 mM MβCD. Also shown are the blots of the IgG isotype control IP probed with EGFR and caveolin-1 antibodies. **B)** Quantification of band intensity of pEGF receptor from three independent experiments. The asterisk indicates *P* < 0.01 by Student’s *t* test.

Caveolin-1 can modulate the turnover of the focal adhesion by binding to components of the focal complex, including the epidermal growth factor (EGF) receptor [34-36]. More specifically, the phosphorylated form of caveolin-1 (caveolin-1 pTyr-14), which is excluded from caveolae, serves as an accessory protein to direct the trafficking of the EGF receptor to the focal complex as well as stabilizes FAK within the complex [37,38]. In essence, the phosphorylated form of caveolin-1 promotes FAK phosphorylation and the turnover of focal complexes [37,39]. Based on the potential interaction between phosphorylated caveolin-1 and the EGF receptor, we performed EGF receptor immunoprecipitation (IP) experiments to determine if phosphorylated caveolin-1 and the EGF receptor are associated in *C. jejuni* infected cells (Figure 4). The experiment revealed that the EGF receptor is activated, as judged by a significant increase in the amount of the pEGF receptor, in *C. jejuni*-infected HeLa cells compared to uninoculated cells. While we predicted the previous result, unexpected was that the treatment of cells with MβCD prevented both the activation of the EGF receptor by *C. jejuni* as well as the association of caveolin-1 with the EGF receptor.

**Figure 5:**
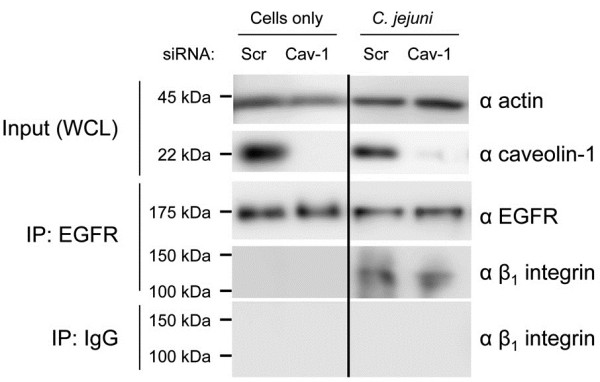
***C. jejuni *induces the association of β**_**1 **_**integrin and EGF receptor (EGFR) independent of caveolin-1.** HeLa cells were treated with caveolin-1 (Cav-1) siRNA and scrambled (Scr) siRNA. The cells were infected with *C. jejuni* and incubated for 30 min. A portion of the whole cell lysate (WCL) was probed for actin (loading control) and caveolin-1 to determine the efficacy of the siRNA treatment (top two blots). The EGFR was then immunoprecipitated (IP) from the WCL, separated by SDS-PAGE, and blotted for EGFR and β_1_ integrin. The EGFR coprecipitated with the β_1_ integrin following *C. jejuni* infection. This association was not disrupted following caveolin-1 knockdown. Also shown is a blot of the IgG isotype control IP probed with an antibody reactive against the β_1_ integrin.

Although it is known that the EGF receptor can be stimulated in the absence of an extracellular ligand via integrin signaling [40], we sought to determine whether phosphorylated caveolin-1 participates in EGF receptor activation. More specifically, we wanted to know if phosphorylated caveolin-1 directs the EGF receptor to the sites containing activated α_5_β_1_-integrins. To address if the association of the EGF receptor with the β_1_ integrin can result in its activation in the absence of phosphorylated caveolin-1, HeLa cells were treated with caveolin-1 siRNA and infected with *C. jejuni.* The EGF receptor antibody was then used for IP experiments. Blots were probed with an antibody reactive against the β_1_ integrin (Figure 5). The blots revealed that the β_1_ integrin co-precipitated with the EGF receptor. Importantly, treatment of the cells with caveolin-1 siRNA resulted in greater than a 90% knockdown in caveolin-1 protein compared with cells transfected with the scrambled siRNA, as judged by immunoblot analysis coupled with densitometry (not shown). These results demonstrated that *C. jejuni* infection of HeLa cells results in the activation of the EGF receptor, via its association with the activated β_1_ integrin, in the presence or absence of phosphorylated caveolin-1.

**Figure 6:**
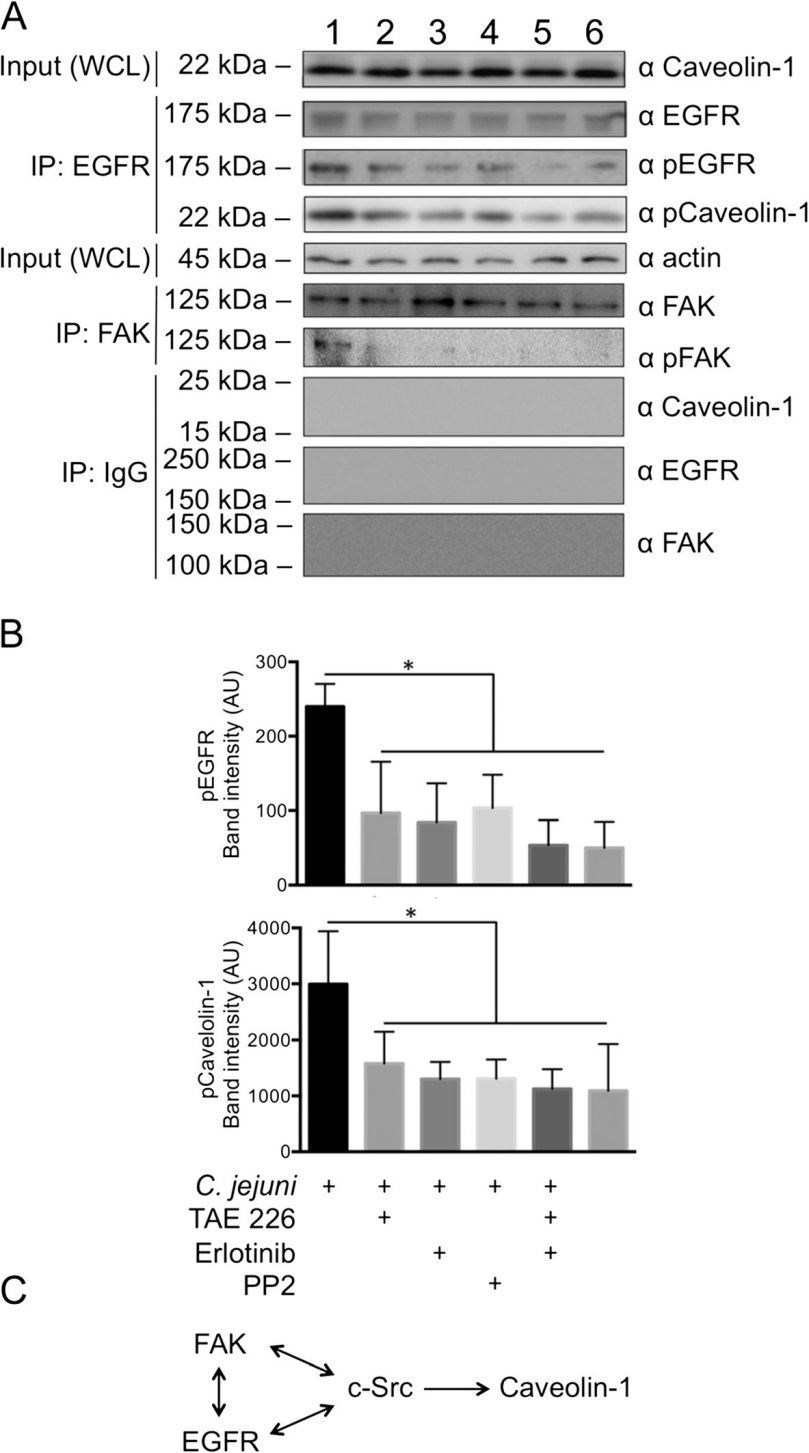
**Treatment of HeLa cells with inhibitors of the EGF receptor (EGFR), FAK, and c-Src activation disrupts *C. jejuni*-dependent EGF receptor and caveolin-1 phosphorylation.** HeLa cells were untreated or treated with inhibitors to FAK (5 μM TAE 226), EGFR (20 μM erlotinib), c-Src (10 μg/ml PP2), and both FAK and EGFR inhibitors, as indicated in the figure. Positive and negative controls consisted of *C. jejuni* infected and uninfected cells. Panels: **A)** Cell lysates were immunoprecipitated with either an EGFR antibody or a FAK antibody, separated by SDS-PAGE, and blotted for total EGFR (loading control), pEGFR, FAK, pFAK, and pCaveolin-1. Lanes: 1, Infected with *C. jejuni* in the absence of any inhibitor (vehicle only); 2, Treated with TAE 226 and infected with *C. jejuni*; 3, Treated with erlotinib and infected with *C. jejuni*; 4, Treated with PP2 and infected with *C. jejuni*; 5, Treated with both TAE 226 and erlotinib and infected with *C. jejuni*; 6, Uninfected cells (Cells only). Also shown are the blots of the IgG isotype control IP probed with caveolin-1, EGFR, and FAK antibodies. **B)** Quantitation of pEGFR and pCaveolin-1 from three independent experiments. The asterisk indicates *P* ≤ 0.05 as judged by one-way ANOVA followed by post-hoc Tukey’s analysis. Each error bar represents ± the standard deviation of the mean (SD). **C)** The data indicates that caveolin-1 is phosphorylated by c-Src, which is stimulated in response to the activation of FAK and the EGFR (Panel **C**).

Only the phosphorylated form of caveolin-1 is associated with components of the focal complex [39]. Given that we observed that the EGF receptor pulls down caveolin-1 by IP (Figure 4), we performed experiments to determine if *C. jejuni* infection of HeLa cells would result in the activation (phosphorylation) of caveolin-1, and if it did, the mechanistic basis of the activation. It is known that caveolin-1 is phosphorylated on Tyr-14 by c-Src [34]. We hypothesized that the activation of the EGF receptor and FAK, which results following *C. jejuni* infection due to the activation of β_1_ integrins, induces c-Src activity and the phosphorylation of caveolin-1. To test our hypothesis, HeLa cells were treated with specific inhibitors of EGF receptor (erlotinib), FAK (TAE 226), and c-Src (PP2) activation. HeLa cells were infected with *C. jejuni,* and EGF receptor IP experiments performed (Figure 6). The blots were then probed with an antibody reactive against the pEGF receptor and phosphorylated caveolin-1. Treatment of *C. jejuni*-infected HeLa cells with the FAK and c-Src inhibitors reduced the total level of the activated (phosphorylated) EGF receptor (Figure 6, Panels A and B). In addition, the level of phosphorylated caveolin-1 associated with the EGF receptor in *C. jejuni-*infected cells was significantly reduced when the cells were treated with drugs that prevented FAK, EGF receptor, and c-Src activity (Figure 6, Panels A and B). Additionally, IP experiments were performed with FAK to demonstrate drug efficacy on inactivation of FAK phosphorylation. Together, these data indicate that the phosphorylation of caveolin-1 occurs via c-Src, which is activated by FAK and the EGF receptor (Figure 6, Panel C). These findings are consistent with our current understanding of the signaling events required to direct proteins to the focal complex [34,37].

### Part III. *C. jejuni* cell invasion is not dependent on caveolin-1

#### *Caveolin-1 is not required for* C. jejuni *invasion of HeLa epithelial cells*

**Figure 7:**
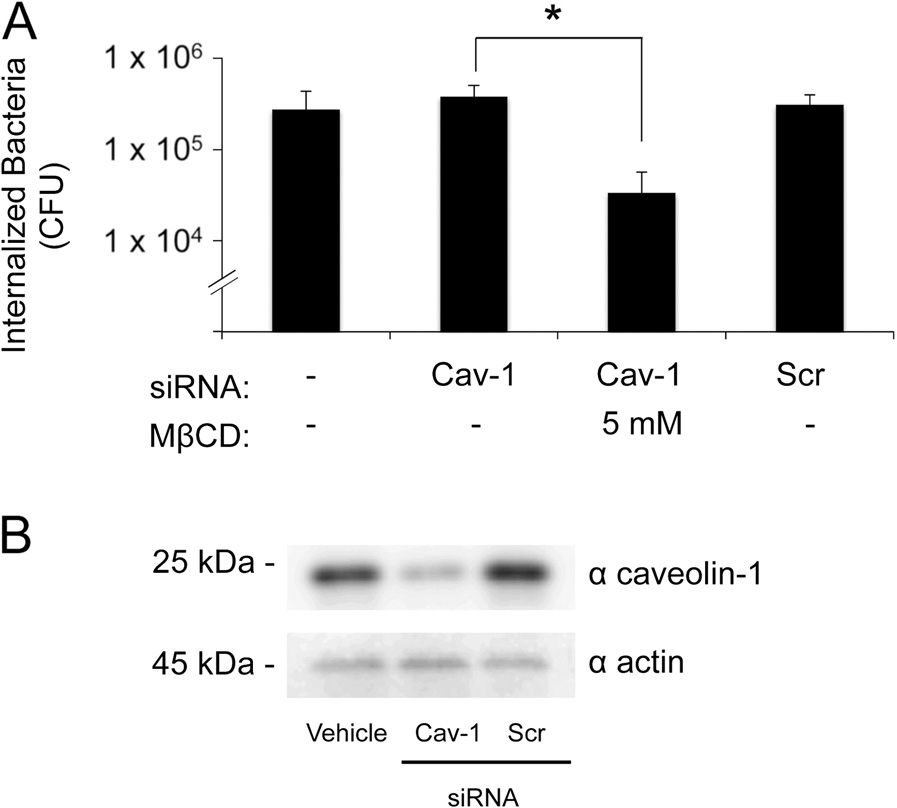
**Knockdown of endogenous caveolin-1 protein does not alter *C. jejuni*-invasion of HeLa cells.** Panels: **A)** Internalization of *C. jejuni* in HeLa cells transfected with caveolin-1 (Cav-1) or scrambled (Scr) siRNA. Also shown is the number of bacteria internalized in HeLa cells transfected with caveolin-1 siRNA and treated with 5 mM of methyl-β-cyclodextrin (MβCD). The values represent the mean number of internalized bacteria ± standard deviation. The asterisks indicate significance between untreated and treated samples (*P* < 0.05), as determined using Student’s *t* test. **B)** Whole cell lysates of untreated (vehicle only), caveolin-1 siRNA transfected, and Scr siRNA transfected HeLa cells probed with an α caveolin-1 antibody. The blot was re-probed with an α actin antibody to confirm that equal amounts of protein were loaded into each well.

**Figure 8:**
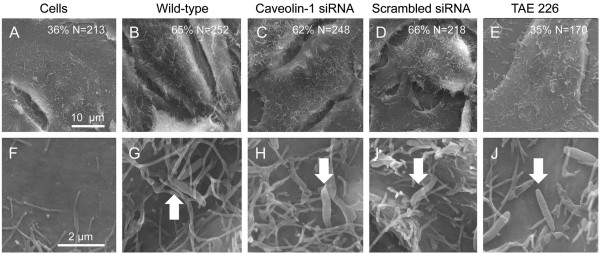
**Knockdown of endogenous caveolin-1 protein does not alter *C. jejuni*-induced membrane ruffling as judged by scanning electron microscopy (SEM).** HeLa cells were transfected with caveolin-1 siRNA or scrambled (Scr) siRNA, infected with *C. jejuni* for 15 min, and processed for examination by SEM as outlined in ‘Methods.’ The images shown are uninfected HeLa cells (Panels **A** and **F**) and *C. jejuni* infected HeLa cells (Panels **B-E** and **G-J**) that are untreated (Panels **B** and **G**), treated with caveolin-1 siRNA (Panels **C** and **H**), scrambled (Scr) siRNA (Panels **D** and **I**), or with the FAK inhibitor TAE 226 (Panels **E** and **J**). Arrows indicate *C. jejuni* associated with the host cells. Also indicated is the percentage of cells with membrane ruffling of total cells (N). In contrast to the *C. jejuni* infected cells in the absence of an inhibitor, *C. jejuni* infected cells treated with caveolin-1 siRNA, and *C. jejuni* infected cells treated with Scr siRNA, treatment of cells with the FAK inhibitor TAE 226 inhibited *C. jejuni*-induced membrane ruffling. The images shown in Panels **A-E** were collected at the same magnification and the images shown in Panels **F-J** were collected at the same magnification.

To further address the contribution of caveolin-1 and caveolae in *C. jejuni* internalization, HeLa cells were treated with caveolin-1 siRNA and scrambled siRNA. The knockdown of caveolin-1 protein in HeLa cells transfected with caveolin-1 siRNA was confirmed by immunoblot analysis coupled with densitometry. Knockdown of caveolin-1 protein in cells had no effect on *C. jejuni* internalization; however, MβCD pretreatment prevented *C. jejuni* internalization in caveolin-1 siRNA treated cells (Figure 7). We then examined the ability of *C. jejuni* to induce membrane ruffling in HeLa cells treated with caveolin-1 siRNA. Focal adhesion kinase (FAK) was also targeted as a control, as FAK is necessary for the downstream signaling events required for *C. jejuni*-induced membrane ruffling and internalization [19]. FAK activation was inhibited by the treatment of HeLa cells with TAE 226. In accordance with the results of the internalization assay, scanning electron microscopy examination revealed that the amount of membrane ruffling induced by *C. jejuni* was indistinguishable in the caveolin-1 siRNA treated versus the untreated HeLa cells (62% versus 65%, respectively) (Figure 8). In contrast, the treatment of HeLa cells with TAE 226 significantly reduced the level of *C. jejuni*-induced membrane ruffling (35% versus 65%, respectively). These data support the hypothesis that *C. jejuni* utilizes components of the focal complex (i.e., FAK) to invade epithelial cells [19]. We concluded that *C. jejuni* invasion of host cells is independent of caveolae based on the following two observations: a) caveolin-1 siRNA treatment of HeLa cells had no effect on *C. jejuni* internalization; and b) caveolin-1 siRNA treatment of HeLa cells did not block *C. jejuni-*induced membrane ruffling.

#### C. jejuni *invade Caco-2 cells*

Caveolin-1 and caveolin-2 are synthesized in a wide variety of tissues [13]. Although their precise function is not clear, caveolae are involved in a number of important cellular processes, including signal transduction, calcium signaling, and endocytosis [9]. To address the question of whether caveolae are required for *C. jejuni* internalization in a different manner, we took advantage of the fact that these structures are not present in all cell types. Since caveolin-1 is essential for caveolae formation, cells lacking this protein do not have caveolae [13]. Consistent with previous reports [7,9,12], a 22 kDa band, corresponding to the *M*_r_ of caveolin-1, was detected in HeLa and INT 407 cells but not in Caco-2 cells, as judged by immunoblot analysis using a caveolin-1 specific antibody (Additional file 7: Figure S7). Others have also been unable to detect caveolin-2 in Caco-2 cells [7,12]. Thus, Caco-2 cells do not have caveolae. We then performed an experiment to examine the effect of MβCD on *C. jejuni* internalization of Caco-2 cells. Interestingly, treatment of Caco-2 cells with MβCD reduced *C. jejuni* internalization in a dose-dependent manner (Additional file 8: Figure S8). These results demonstrate that MβCD inhibits *C. jejuni* internalization regardless of whether the cells possess caveolae structures/caveolin-1. Noteworthy is that MβCD has been reported to disrupt all lipid rafts [41].

Transfection of Caco-2 cells with a plasmid that expresses caveolin-1 cDNA results in the proper localization of caveolin-1 and the formation of caveolae [9]. Based on the sum of the data, the investigators concluded that caveolin-1 in Caco-2 cells behaves similar to caveolin-1 expressed in cells that normally express the protein [9]. To determine if the presence of caveolae might potentiate the number of *C. jejuni* internalized, binding and internalization assays were performed with Caco-2 cells that were transfected with a plasmid expressing caveolin-1 (Additional file 9: Figure S9). No difference was observed in the number of *C. jejuni* internalized in Caco-2 expressing caveolin-1 versus the control cells (i.e., Caco-2 cells transfected with a plasmid without an insert). Importantly, caveolin-1 was detected only in the Caco-2 cells transfected with the caveolin-1 containing plasmid, as judged by immunoblot analysis with a caveolin-1 specific antibody.

An alternative approach to expressing caveolin-1 in cells that normally do not express the protein and have no caveolae is to use caveolin-1 knockout cells. More specifically, the 3T3 mouse embryonic fibroblast knockout cell line (3T3 MEF KO, CRL-2753) is homozygous for disruption of the caveolin-1 gene (Cav-1^-/-^) whereas the 3T3 MEF wild-type cell line (3T3 MEF WT, CRL-2752) is Cav-1^+/+^. We performed *C. jejuni* binding and internalization with the 3T3 MEF WT and 3T3 MEF KO cells (Additional file 10: Figure S10). We did not observe a difference in the numbers of *C. jejuni* bound to or internalized in the 3T3 MEF WT versus the 3T3 MEF KO cells. Taken together, these results are consistent with the proposal that *C. jejuni* invasion of host cells occurs in a caveolae-independent manner.

#### MβCD treatment of HeLa cells disrupts β_1_ integrin and EGF receptor association

**Figure 9:**
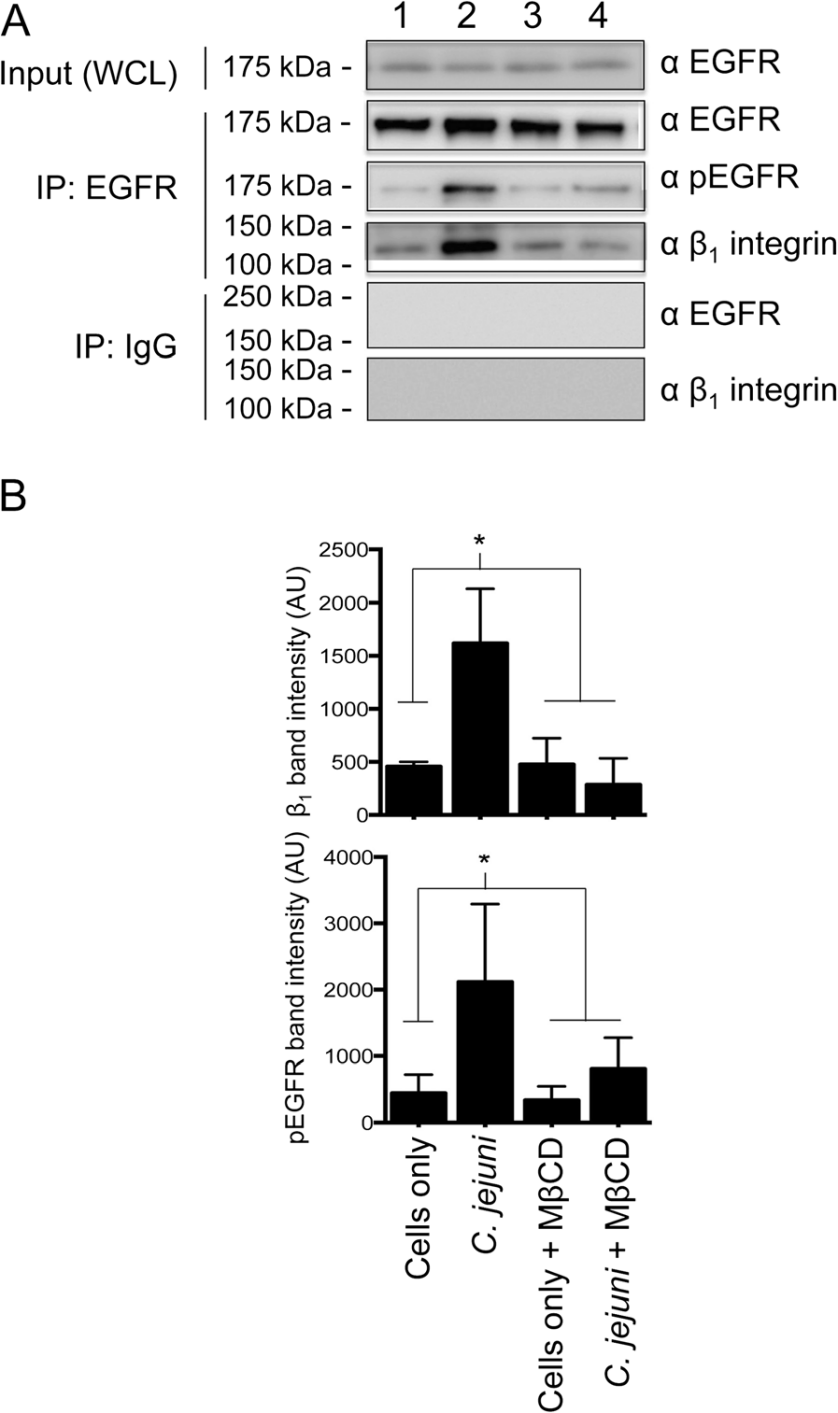
**Treatment of HeLa cells with methyl-β-cyclodextrin (MβCD) disrupts the *C. jejuni*-dependent association of phospho-EGF receptor (pEGFR) with β**_**1 **_**integrin.** HeLa cells were infected with *C. jejuni* in the presence and absence of 5 mM MβCD for 45 min. Panels: **A)** Cell lysates were immunoprecipitated with an EGFR antibody, separated by SDS-PAGE, and blotted for β_1_ integrin, pEGFR, and total EGFR (loading control). HeLa cells were uninfected (Lanes 1 and 3) or *C. jejuni* infected (Lanes 2 and 4) in the absence (Lanes 1 and 2) or presence of MβCD (Lanes 3 and 4). Also shown are the blots of the IgG isotype control IP probed with an antibodies reactive against the EGFR and the β_1_ integrin. **B)** Quantification of band intensity of β_1_ integrin and pEGFR from three independent experiments. The asterisk indicates *P* < 0.01 by one-way ANOVA followed by post-hoc Dunnet’s analysis.

To address the findings of previous reports, we performed experiments to determine the mechanistic basis for MβCD inhibition of *C. jejuni* internalization. We hypothesized that MβCD prevents the host signaling response triggered by *C. jejuni* infection, thereby resulting in a decrease in internalized bacteria, based on the following observations: a) treatment of cells with MβCD disrupts all lipid rafts [41]; b) a substantial amount of the EGF receptor is localized to lipid rafts, but not to caveolae [42]; c) activation of the EGF receptor (phosphorylation of residues Tyr-845, Tyr-1068, Tyr-1086 and Tyr-1173) can be activated in the absence of EGF by its association with the β_1_ integrin (ligand independent activation) [40]; and d) β_1_ integrin localization (raft-mediated endocytosis) is sensitive to cholesterol depletion (i.e., MβCD treatment of cells) [43]. Noteworthy is that we have shown that *C. jejuni* infection of cells results in β_1_ integrin and EGF receptor association (Figure 5) and EGF receptor activation (Figure 4). Thus, we specifically tested if MβCD treatment of HeLa cells disrupts β_1_ integrin and EGF receptor association. The results of this experiment revealed that MβCD completely inhibited EGF receptor activation and disrupted the association of the β_1_ integrin and EGF receptor, as judged by IP experiments (Figure 9). HPβCD treatment of HeLa cells also inhibited EGF receptor activation and disrupted the association of the β_1_ integrin and EGF receptor (Additional file 11: Figure S11), and MβCD treatment of Caco-2 cells (caveolin-1 negative) prevented EGF receptor activation (Additional file 12: Figure S12). These findings provide a plausible explanation for the inhibitory effect of MβCD on *C. jejuni* internalization.

## Discussion

Caveolae are flask-like structures that are enriched in cholesterol and glycosphingolipids; however, caveolae are different from planar lipid rafts based on the presence of the cholesterol-binding protein caveolin-1. Caveolin-1 appears to stabilize the invaginated structure of caveolae. Regardless of the specific type of lipid raft, these regions provide a spatial and temporal meeting site for signaling molecules, thus serving as signaling platforms [8].

A number of bacteria, including *Campylobacter jejuni* and *Salmonella enterica* serotype Typhimurium, have been proposed to require caveolae for cell invasion based on the observation that treatment of cells with filipin III or MβCD blocked bacterial internalization in a dose-dependent manner [15,17,44]. Wooldridge *et al.*[15] observed a dose-dependent reduction in *C. jejuni* invasion of Caco-2 cells when the cells were treated with filipin III, and concluded that caveolae are involved in *C. jejuni* uptake. Subsequently, Watson and Galan [45] reported that the treatment of human T84 cells with MβCD blocked *C. jejuni* internalization. However, based on additional assays, Watson and Galan [45] speculated that *C. jejuni* internalization might not be associated with caveolae-mediated endocytosis, but that that caveolae or caveolin-1 may play a role in the host cell signaling events necessary for bacterial uptake. Our data indicate that *C. jejuni* internalization occurs in a caveolae-independent manner. We did find that treatment of HeLa cells with MβCD and HPβCD, two cholesterol-depleting compounds, reduced *C. jejuni* internalization in a dose-dependent manner. These results support the hypothesis that *C. jejuni* internalization occurs in a caveolae-dependent manner, as caveolin-1 is a cholesterol binding protein and the treatment of cells with a cholesterol-depleting compound would disrupt the cellular localization of caveolin-1. However, knockdown of caveolin-1 protein in HeLa cells by treatment with caveolin-1 siRNA had no effect on *C. jejuni* internalization. In addition, treatment of Caco-2 cells with MβCD inhibited *C. jejuni* internalization (Additional file 8: Figure S8). Caco-2 cells do not express caveolin-1 and, as a consequence, do not have caveolae (Additional file 7: Figure S7) [9,12]. We expressed caveolin-1 in Caco-2 cells to attempt to enhance the invasiveness of *C. jejuni*, but did not observe a change in the number of *C. jejuni* internalized versus Caco-2 cells transfected with a plasmid without an insert (caveolin-1 negative cells) (Additional file 9: Figure S9). Consistent with this finding, the number of *C. jejuni* bound to and internalized in 3T3 MEF Cav-1^+/+^ cells was not different from that of 3T3 MEF Cav-1^-/-^ cells (Additional file 10: Figure S10). We further demonstrated that MβCD and HPβCD disrupts the interaction between EGF receptor and the β_1_ integrin, which explains the mechanistic basis for the inhibition of *C. jejuni* internalization with this inhibitor (Figure 9 and Additional file 11: Figure S11). Taken together, our findings show that MβCD does not specifically target and disrupt the function of caveolae; it disrupts all lipid rafts and blocks the outside-in signaling that *C. jejuni* induces from binding to fibronectin. Also worth mentioning is that caveolin-containing vesicles do not contain markers of early endosomes, such as the early endosome marker EEA-1, lysosomes, ER or Golgi [46,47]. However, researchers have found that the *Campylobacter*-containing vacuoles (CCVs) contain markers of both early and late endosomes [45,48]. More specifically, CCVs display EEA-1, and then acquire the Rab5 and Rab7 GTPases as well as the lysosomal-associated membrane protein 1 (LAMP-1). We found that *C. jejuni* in the process of internalization are associated with the cell adhesion proteins paxillin and vinculin (Additional file 5: Figure S5 and Additional file 6: Figure S6), which is consistent with the mechanism of receptor-mediated endocytosis. In summary, we show that the long-standing conclusion that *C. jejuni* invades via caveolae is inaccurate.

Nethe and Hordijk [34] concluded that caveolin-1, in general, serves as a negative regulator of cell signaling. However, the phosphorylated form of caveolin-1 (caveolin-1 pTyr-14), which is excluded from caveolae, can interact with various components of the focal complex, including FAK, the β_1_ integrin, and phosphorylated paxillin [39]. The phosphorylated from of caveolin-1 is also known to stabilize the localization of FAK within the focal complex resulting in the recruitment of p130Cas and paxillin that promote focal complex turnover [37].

Our data indicate that the integrins and EGF signaling pathway act in a cooperative manner to promote *C. jejuni* internalization. Although the EGF receptor is activated by binding of an extracellular ligand, it can be activated in the absence of an extracellular ligand via integrin signaling. In the case of integrin-dependent EGF receptor-phosphorylation, activation is dependent upon a multimeric complex of integrins, c-Src, p130Cas and the EGF receptor [40]. Activation of the EGF pathway alters components of the cytoskeleton involved with actin organization, focal adhesion formation and resolution, as well as cell-cell adhesion [49]. We had previously found that pre-treatment of INT 407 cells with PD168393 and erlotinib, which are specific inhibitors of EGF receptor tyrosine phosphorylation, significantly inhibited *C. jejuni-*mediated host cell membrane ruffling and invasion [19]. We had also found that the EGF receptor becomes phosphorylated at sites Tyr-845 and Tyr-1068 upon infection with *C. jejuni*, which is consistent with the idea that the EGF receptor is stimulated via integrin signaling (residues Tyr-845, Tyr-1068, Tyr-1086 and Tyr-1173) [40]. Noteworthy is that pre-treatment of INT 407 cells with EGF rescues the invasiveness of a non-invasive *C. jejuni* mutant [19]. Here we report that infection of HeLa cells with *C. jejuni* induces EGF receptor activation, as evidenced by an increase in the level of phosphorylated EGF receptor when compared to uninfected cells. We have also found that pre-treatment of HeLa cells with MβCD disrupts *C. jejuni*-dependent EGF receptor phosphorylation. Our findings are consistent with reports showing that the EGF receptor is localized in lipid rafts, but not in caveolae [42,50].

To dissect the relationship between Rac1 activity and the involvement of actin in *C. jejuni* internalization, HeLa cells were treated with cytochalasin D prior to infection. Cytochalasin D depolymerizes actin filaments by binding to the (+) end of F-actin, thereby blocking the addition of G-actin subunits to these sites [51]. We found that the treatment of HeLa cells with cytochalasin D had no affect on Cia protein delivery or Rac1 activation (which must occur prior to microfilament re-organization), but prevented *C. jejuni*-induced membrane ruffling (Figures 2 and 3). This finding is consistent with previous work demonstrating the involvement of microfilaments (MFs) in *C. jejuni* internalization [20] and our model in which we propose that *C. jejuni*-host cell contact and the delivery of the Cia proteins to the host cell cytosol promotes the activation of focal complex components and Rac1.

While previous studies have shown that *C. jejuni* internalization is sensitive to microtubule inhibitors [52,53], the role of microtubules (MTs) in *C. jejuni* invasion has not been clear. To gain insight into the potential role of microtubules in *C. jejuni* invasion, we treated HeLa cells with nocodazole. This drug binds β-tubulin, preventing tubulin polymerization [25]. We found that the treatment of HeLa cells with nocodazole had no affect on Cia protein delivery, but prevented both Rac1 activation and *C. jejuni*-induced membrane ruffling (Figures 2 and 3). One possible reason that nocodazole treatment reduces *C. jejuni* internalization is that MTs and MFs act cooperatively in recycling integrin receptors [54,55]. Alternatively, nocodazole could reduce the number of *C. jejuni* internalized by preventing the disassembly of focal adhesions [56]. Importantly, focal complexes and focal adhesions are two related types of cellular attachment complexes that differ in their molecular composition and the rate of association versus dissociation of their molecular components. Focal complexes are transient attachments that form at the tip of a cellular protrusion whereas focal adhesions are larger, longer-lived attachments from which stress fibers organize. Treatment of cells with low concentrations of nocodazole results in enlarged focal adhesions. Moreover, nocodazole reduces Rac1 membrane targeting and activation, thereby slowing the turnover of focal complexes [8,54,57]. We favor a model whereby the treatment of HeLa cells with nocodazole reduces the total number of dynamic structures that are involved in *C. jejuni* internalization (focal complexes), which in turn inhibits Rac1 recruitment, Rac1 activation, actin rearrangement, and membrane ruffling. In essence, nocodazole and MβCD inhibit the cell signaling events necessary to initiate cytoskeletal rearrangement, whereas cytochalasin D blocks cytoskeletal rearrangement by preventing actin polymerization.

## Conclusion

**Figure 10:**
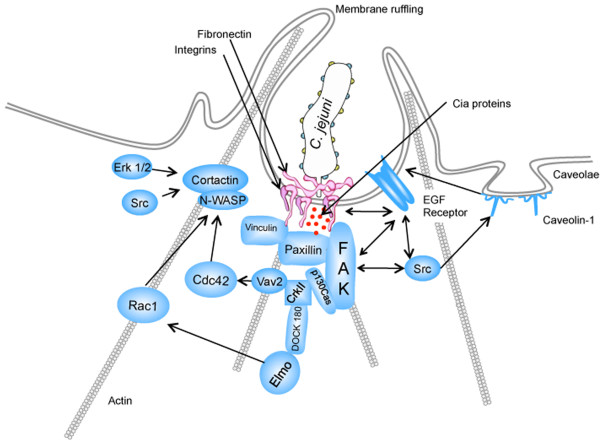
**Comprehensive model of *C. jejuni *internalization.** The activation of the EGF receptor and FAK, which results following *C. jejuni* infection via the activation of the α_5_β_1_ integrins, induces c-Src activity and the phosphorylation of caveolin-1. Uptake of *C. jejuni* requires the recruitment of key components of the focal complex (i.e., FAK, paxillin, vinculin, p130Cas, the CrkII/DOCK-180/ELMO complex). Focal complex assembly promotes the activation of Erk 1/2, Rac1, Cdc42, cortactin, and N-WASP leading to actin cytoskeletal reorganization.

Based on published data and the findings herein, a refined model of *C. jejuni* invasion can be generated where the internalization of *C. jejuni* by epithelial cells is dependent upon components of the focal complex and involves cholesterol-rich (non-caveolae) lipid raft domains in the host plasma membrane (Figure 10). Activation of the α_5_β_1_ integrins by *C. jejuni* binding to fibronectin results in outside-in signaling, resulting in FAK and EGF receptor activation. The activation of FAK promotes paxillin and Src activation. The activation of the EGF receptor and Src results in the phosphorylation of caveolin-1, which associates with the EGF receptor. Paxillin serves as a point of convergence, where integrin-dependent signals from the ECM trigger intracellular signal transduction involved in actin rearrangement. The phosphorylation of paxillin at Tyr-31 and Tyr-118 by the FAK-Src complex creates binding sites on paxillin for p130Cas and the Crk family of adaptor proteins, which in turn activate Rac1 via the CrkII/DOCK180/ELMO complex. Noteworthy is that a *C. jejuni* CiaC secreted protein is, in part, responsible for Rho GTPase Rac1 recruitment and activation, as judged by immuno-fluorescence microscopy and activated Rac1 G-LISA™, respectively. We have also shown that Erk 1/2 and cortactin activation are required for membrane ruffling downstream of activated Rac1 [58,59]. Our data shows that caveolin-1 is not required for *C. jejuni* internalization, but it is associated with components of the focal complex following *C. jejuni*-induced activation of the EGF receptor and FAK via α_5_β_1_ integrin receptor stimulation. Our data further indicate that activation of FAK and the EGF receptor triggers the activation of c-Src, which then acts to phosphorylate caveolin-1. Finally, we present a plausible explanation for why MβCD, cytochalasin D, and nocodazole treatment of epithelial cells reduces the internalization of *C. jejuni*. Together, these findings provide new insight into the mechanism that *C. jejuni* utilizes to invade epithelial cells.

## Methods

### Bacterial strains

The *C. jejuni* wild-type F38011 strain was grown in Mueller-Hinton (MH) broth, on MH agar plates containing 5% citrated bovine blood (MH-blood), or in biphasic cultures (10 ml of MH broth overlaid on a MH agar plate) in a microaerobic atmosphere (5% O_2_, 10% CO_2_, 10% H_2_, 75% N_2_).

### Tissue culture

HeLa (ATCC® CCL2™), Caco-2 (ATCC® HTB-37™), 3T3 MEF WT (ATCC® CRL-2752™), and 3T3 MEF KO (ATCC® CRL-2753™) cells were purchased from the American Type Culture Collection (Manassas, VA) and were grown in Minimal Essential Medium (MEM) supplemented with 10% (vol/vol) fetal bovine serum (FBS, Hyclone Laboratories Inc., Logan, UT) and 5% L-glutamine (1.8 mM). The cells were incubated at 37°C in a humidified, 5% CO_2_ incubator, and passaged every 48 to 72 h.

### Inhibitors

The stock inhibitors used in this study were prepared as indicated. Methyl-β-cyclodextrin (MβCD) (500 mM), HPβCD (500 mM), and erlotinib (20 mM) were prepared in water. Filipin III (1 mM), nystatin (50 mg/ml), nocodazole (2 mM), cytochalasin D (100 mM), and PP2 (10 mg/ml) were prepared in DMSO. TAE 226 (1 mM) was prepared in methanol.

### *C. jejuni*-cell infection assays

*C. jejuni* binding and internalization assays were performed with HeLa, Caco-2, and 3T3 MEF cells as outlined previously [60]. All assays were performed at a multiplicity of infection (MOI) ranging between 50 and 500, and repeated a minimum of 3 times to ensure reproducibility. The reported values represent the mean counts ± standard deviations derived from quadruplicate wells.

To test the effect of MβCD, HPβCD, filipin III, and nystatin on *C. jejuni* cell invasion, HeLa cells were pre-treated for 30 min in MEM containing a range of concentrations of the inhibitors. Following incubation, a suspension of *C. jejuni* in MEM was added to each well and binding and internalization assays were performed using standard laboratory protocols. To determine if an inhibitor or the vehicle (water or DMSO) had an effect on the viability of HeLa cells, the cells were rinsed twice with PBS following inhibitor treatment, stained with 0.5% trypan blue for 5 min, and visualized with an inverted microscope. To determine the specificity of MβCD treatment, cholesterol was restored to the membrane as described previously [61]. Briefly, cyclodextrin:cholesterol complex was formed at a cyclodextrin:cholesterol molar ratio of 8:1. The HeLa cells were treated with 5 mM MβCD for 30 min and then the cyclodextrin:cholesterol complex was added at 5 mM for an additional 30 min prior to infection with *C. jejuni*.

### Scanning electron microscopy

Scanning electron microscopy was performed as described previously [19]. Briefly, HeLa cells were pre-treated with MβCD (5 mM), nocodazole (20 mM), and cytochalasin D (1 mM), for 30 minutes prior to inoculation with *C. jejuni*. EGF (100 ng/ml) was added to cells 5 min prior to inoculation with *C. jejuni*. Quantification of membrane ruffling was done by two independent observers and tabulated. Only cells with clear boundaries were counted and cells positive for membrane ruffling were scored.

### Adenylate cyclase domain (ACD) reporter delivery assays

The delivery of the *C. jejuni* CiaC protein to HeLa cells was determined using laboratory established protocols [27]. We used the adenylate cyclase domain (ACD) of the *Bordetella pertussis* CyaA protein fused to the *C. jejuni* CiaC protein as a reporter for protein delivery to host cells [62-64]. ACD catalyzes the production of cAMP from ATP only in the presence of the host cell protein calmodulin. As a negative control, the *metK* gene driven from its native promoter was also cloned in-frame with the ACD. The *C. jejuni* MetK protein is a cytosolic *S*-adenosylmethionine synthetase [29]. Briefly, HeLa cells were pre-treated with MβCD (5 mM), nocodazole (20 mM), cytochalasin D (1 mM), TAE 226 (5 μM), and DMSO (vehicle only control) for 30 minutes prior to inoculation with *C. jejuni*. The amount of intracellular cAMP produced in HeLa cells was assayed at 30 min post-infection by ELISA (Assay Designs, Farmingdale, NY). The assay was repeated 5 times to ensure reproducibility.

### Assessment of Rho GTPase Rac1 activation

HeLa cells were seeded into 6 well tissue culture plates at a density of 2 × 10^5^ cells/well and serum starved for 24 hours. Cells were pre-treated with vehicle (DMSO), MβCD (5 mM), nocodazole (20 mM), and cytochalasin D (1 mM) for 30 minutes prior to inoculation with *C. jejuni* resuspended in PBS. The amount of activated Rac1 in *C. jejuni*-infected and uninfected cells was determined using the G-LISA™ Rac1 Activation Assay according to the manufacturer’s instructions (Cytoskeleton, Denver, CO).

### Transfection of cells with small interfering RNA (siRNA) or plasmids

HeLa cells were transfected with small interfering RNA (siRNA) using lipofectamine RNAiMax (Invitrogen, Grand Island, NY) according to the manufacturer’s instructions. Caveolin-1 stealth siRNA (Invitrogen, 21244939) and scrambled control siRNA (Santa Cruz Biotechnology, Inc., CA, sc-37007) were applied to the cells 24 h before the assay. A standard binding and internalization assay was then performed. Knockdown of endogenous proteins was confirmed by immunoblot with antibodies for specific host proteins. Caco-2 cells were transfected with plasmids encoding for caveolin-1-eGFP or the empty vector control eGFP using Lipofectamine LTX and Plus**™** Reagent according to manufacturer’s instructions (Invitrogen, 15338–100)

### Immunoprecipitation

HeLa cells were seeded at 3 × 10^6^ cells/well and serum starved in MEM for 3 h prior to the addition of *C. jejuni* with and without treatment as well as an uninfected (negative control). Cells were pretreated 30 min prior to and during *C. jejuni* infection with either 10 μg/ml PP2, 20 μM erlotinib, and/or 5 μM TAE 226, or with 5 mM MβCD added at the time of infection. At 45 min following infection, cells were collected in ice-cold lysis buffer [10 mM Hepes, 10% glycerol, 50 mM sodium fluoride, 150 mM sodium chloride, 1% Triton X-100, 20 μg/ml DNAse (Sigma, D5025), Protease Inhibitor Cocktail (Sigma, P2714), 1 mM phenylmethylsulfonyl fluoride (PMSF), and 1 mM sodium orthovanadate]. Immunoprecipitations were performed by incubating cell lysates with either an anti-FAK antibody (610087, BD Biosciences, San Jose, CA) or an anti-EGF receptor mAb 528 antibody (Santa Cruz, sc-120) at 4°C overnight and then adding protein A/G beads at 4°C for 1 h with rotation. The bead complexes were washed and dissolved in sample buffer. Blots were probed with an antibody that reacts against the active forms of FAK (pFAK Y-925, Cell Signaling, 3284) and the EGF receptor (pEGFR Y-1068, Cell Signaling Technology, Inc., Danvers, MA). Blots were also probed with an antibody that reacts against FAK (Santa Cruz, 557), the EGF receptor (Santa Cruz Biotechnology, sc-03), caveolin-1 (Cell Signaling Technology, Inc., Danvers, MA), and pCaveolin-1 (BD Biosciences). The reactive bands were detected using the enhanced chemiluminescence (ECL) Prime system (GE Healthcare Biosciences, Piscataway, NJ) coupled with a GE LAS-4000 mini. Densitometry was performed using Fujifilm Multi Gauge software (Fujifilm, Valhalla, NY). The intensity of each band was obtained by subtracting the average background from adjacent areas in each lane from the total level of the appropriate molecular weight. Each of these values was then normalized to the cells only negative control.

### Preparation of whole cell lysates

Whole cell lysates (WCLs) of HeLa and Caco-2 cells were prepared by the addition of lysis buffer, as described above. The lysates were collected and analyzed by SDS-PAGE coupled with immunoblot analyses. The protein concentration of each supernatant was determined by the bicinchoninic acid (BCA, Pierce, Rockford, IL) protein assay and normalized prior to performing SDS-PAGE.

### SDS-polyacrylamide gel electrophoresis (PAGE) and immunoblot analysis

WCLs were subjected to SDS-PAGE and transferred to polyvinylidene fluoride membranes (PVDF; Immobilon P^SQ^; Millipore Corp., Bedford, MA) for immunoblot analysis [65]. The following antibodies were used for immunoblot analysis: goat α actin polyclonal antibody (Santa Cruz, sc-1616), rabbit α caveolin-1 XP® monoclonal antibody (Cell Signaling Technology, 3267), mouse α caveolin-1 (pTyr-14) (BD Transduction Laboratories, 611338), rabbit α EGF receptor polyclonal antibody (Santa Cruz, sc-03), rabbit α phospho-EGF receptor (Tyr-1068) monoclonal antibody, (Cell Signaling Technology, 3777), rabbit α FAK (Santa Cruz, sc-557), rabbit α phospho-FAK (Y925) (Cell Signaling Technology, 3284) and rabbit α β_1_ integrin polyclonal antibody (Cell Signaling Technology, 4706). The secondary antibodies used in this study were: goat α rabbit IgG (Sigma, A6154), rabbit α goat IgG (Sigma, A5420), and goat α mouse IgG (Sigma, A4416).

### Immunofluorescence microscopy

HeLa cells were plated on 22 mm^2^ glass coverslips at 50% confluence and cultured overnight, and then treated with either 5 mM of MβCD or solvent without drug for 75 minutes. The 75 min time point encompasses the normal assays that used a 30 min pre-treatment with inhibitor and the 45 min infection period with *C. jejuni* and the inhibitor. Cells were rinsed with PBS, fixed with 4% methanol-free paraformaldehyde (Pierce Biotechnology, Inc, #PI-28906) in PBS for 20 minutes, rinsed, and then incubated with a rabbit polyclonal anti-caveolin antibody (BD Biosciences #610059) at a dilution of 1:100 in PBS containing 0.1% Tween 20 and 0.02 mg/ml sodium azide (PBS-TW) for three hours at room temperature. After rinsing in PBS-TW, the cells were incubated for two hours at room temperature with FITC labeled goat anti-rabbit secondary antibody (Jackson ImmunoChemicals #111-096-047) at a dilution of 1:200 in PBS TW containing 0.025 mg/ml rhodamine-labeled phalloidin (SigmaAldrich #P-1951). After a final rinse, coverslips were mounted in medium consisting of 90% glycerol, 10% 10X PBS and 1% DABCO and preparations sealed with nail polish. Cells were viewed with a Leica SP5 confocal microscope using a 63X 1.4 NA objective lens and a pinhole set to an Airy unit size of 1 and a pixel size of 60 nm. Detector gain settings were optimized for imaging of control cells and left at this setting for imaging of MβCD treated cells. Images shown are single confocal sections and are representative of at least three full field images of cell monolayers showing more than 100 cells per image.

HeLa cells were incubated with *C. jejuni* for 45 min at 37**°**C in a 5% CO_2_ incubator prior to fixing with 3.7% paraformaldehyde for 15 min. *C. jejuni* were stained with a 1° rabbit α *C. jejuni* antibody and a 2° Texas Red dye-conjugated donkey α-rabbit antibody (Jackson Immunoresearch Labs, West Groves, PA). Paxillin and vinculin were detected using a mouse α paxillin polyclonal antibody (Product #610052, BD Transduction Laboratories™) and a mouse IgG1 α vinculin monoclonal antibody (Product #V9131, Sigma) followed by incubation with a goat IgG (H + L)-FITC labeled antibody (Boehringer Mannheim, Indianapolis, IN). The coverslips were mounted with 90% glycerol plus 10% 10X PBS containing 1, 4-Diazabicyclo[2.2.2]octane (DABCO™) as an antifading agent. Images were obtained using a Leica TCS SP5 confocal microscope using a 63X, 1.4 NA oil immersion objective lens. All experiments were repeated a minimum of three times and at least 5 fields of view were observed to ensure reproducibility. The quantification of bacteria co-localization was performed by assessing the number of bacteria that were in direct contact with host cells (touching or within the defined borders of the HeLa cells). Co-localization was defined as any *C. jejuni* within the cell border that is in contact with paxillin or vinculin. The interaction between cell-associated *C. jejuni* and focal complexes was performed from six randomly selected fields of cells in a total of two trials were scored by a blinded investigator.

### Statistical analysis

All data was evaluated using a Student’s *t* test or one-way ANOVA followed by post-hoc Tukey’s analysis of the means, using Prism 6 (GraphPad Software, La Jolla, CA). Statistical significance was defined by a value of *P < 0.05.

## Abbreviations

AU: Arbitrary unit; ACD: Adenylate cyclase domain; Cia: *Campylobacter* invasion antigens; DMSO: Dimethyl sulfoxide; DN: Dominant-negative; ECM: Extracellular matrix; EGF: Epidermal Growth Factor; FAK: Focal adhesion kinase; HPβCD: 2-hydroxypropyl-β-cyclodextrin; LB: Luria-Bertani; MβCD: Methyl-β-cyclodextrin; MEM: Minimal essential medium; MH: Mueller-Hinton; MFs: Microfilaments; MTs: Microtubules; MOI: Multiplicity of infection; PVDF: Polyvinylidene fluoride; T3SS: Type III Secretion System; WCLs: Whole cell lysates.

## Competing interests

The authors declare that they have no competing interests.

## Authors' contributions

Conceived and designed the experiments: MEK, DRS, TPE, EAS, and JLO. Performed the experiments: MEK, DRS, TPE, EAS, and JLO. Analyzed the data: MEK, DRS, TPE, EAS, and JLO. Contributed reagents/materials/analysis tools: MEK, DRS, TPE, EAS, and JLO. Contributed to writing the paper: MEK, DRS, TPE, EAS, and JLO. All authors read and approved the final manuscript.

## Supplementary Material

Additional file 1: Figure S1 *C. jejuni* internalization into HeLa cells treated with methyl-β-cyclodextrin (MβCD) is recovered by cholesterol replenishment. HeLa cells were treated with 5 mM MβCD for 30 min. Membrane cholesterol was then replenished by treatment with cyclodextrin:cholesterol complex for 30 min prior to infection with *C. jejuni*. Panels: A) Host cell association was unaffected by MβCD treatment and cholesterol replenishment. B) Treatment with 5 mM MβCD significantly reduced *C. jejuni* internalization. Membrane cholesterol restoration through cyclodextrin:cholesterol complex treatment recovered the invasion phenotype. The asterisk indicates a significant reduction in *C. jejuni* internalization compared to cells infected with *C. jejuni* in the absence of the inhibitor (vehicle), as judged by one-way ANOVA followed by post-hoc Dunnets’s analysis (*P* < 0.05). Each error bar represents ± the standard deviation of the mean (SD). Click here for file

Additional file 2: Figure S2 Effects of methyl-β-cyclodextrin (MβCD) treatment on caveolin-1 localization in HeLa cells. Cells shown were labeled with anti-caveolin primary and fluorescein labeled secondary antibodies (green) and counterstained with rhodamine-labeled phalloidin to visualize filamentous actin (red). Vehicle treated cells (Panels A and C) show abundant caveolin-1 in the vicinity of the cell boundary. Caveolin-1 was localized at or proximal to the cytoplasmic boundary of the actin cytoskeleton underlying the cell plasma membrane (inset in Panel A and arrows in Panel C), suggesting the caveolin-1 may be in the process of recycling. In contrast, MβCD treated cells displayed fewer and smaller sites of caveolin-1 staining (Panels B and D), and appeared more closely associated with actin at the cell boundary (D). Bars are 10 microns (Panels A and B) and 2 microns (Panels C and D). Click here for file

Additional file 3: Figure S3 Treatment of HeLa cells with the cholesterol-depleting compound hydroxypropyl-β-cyclodextrin (HPβCD) reduces *C. jejuni* internalization. HeLa cells were treated with 2.5, 5.0, 7.5, 10, and 20 mM of HPβCD for 30 min prior to inoculation with *C. jejuni*. The control consisted of cells infected with *C. jejuni* in the absence of the inhibitor in medium containing vehicle (i.e., water). Bars indicate the number of adherent (Panel A) and internalized (Panel B) bacteria. The asterisk indicates a significant reduction in *C. jejuni* internalization compared to cells infected with *C. jejuni* in the absence of the inhibitor in medium alone, as judged by one-way ANOVA followed by post-hoc Dunnets’s analysis (*P* < 0.05). Each error bar represents ± the standard deviation of the mean (SD). Click here for file

Additional file 4: Figure S4 Treatment of cells with the cholesterol binding agents filipin III or nystatin had no effect on *C. jejuni* internalization. HeLa cells were treated with a range of concentrations of filipin III (Panel A) and nystatin (Panel B) for 30 min prior to inoculation with *C. jejuni*. The control consisted of cells infected with *C. jejuni* in the absence of the inhibitor in medium containing vehicle (i.e., DMSO) (Panel C). Each error bar represents ± the standard deviation of the mean (SD). Click here for file

Additional file 5: Figure S5 Methyl-β-cyclodextrin (MβCD) treatment of cells reduces the co-localization of *C. jejuni* with the focal complex components paxillin and vinculin. HeLa cells were infected with *C. jejuni* in the absence (Panels A and B) or presence of MβCD (Panels C and D) and examined by confocal microscopy. Paxillin (Panels A and C) and vinculin (Panels B and D) are shown in blue and *C. jejuni* is shown in red. Also shown is an increased magnification of the image (insert). Sites of co-localization observed in a given field are indicated (arrows). In total, 42.0% of cell-associated *C. jejuni* were co-localized with paxillin (N = 71 of 169) and 40.3% of cell-associated *C. jejuni* were co-localized with vinculin (N = 64 of 159 total). Following treatment with MβCD, 25.4% of cell-associated *C. jejuni* were co-localized with paxillin (N = 33 of 130) and 24.7% of cell-associated *C. jejuni* were co-localized with vinculin (N = 22 of 89 total). Scale bar is 10 microns for low magnification images and 2 microns for the higher magnification images. Click here for file

Additional file 6: Figure S6 Additional confocal microscopy images showing *C. jejuni* associated with paxillin and vinculin. Paxillin (Panels A-C) and vinculin (Panels D-F) are shown in blue and *C. jejuni* is shown in red. Also shown is an increased magnification of each image (insert). Scale bar is 10 microns for low magnification images and 2 microns for the higher magnification images. Click here for file

Additional file 7: Figure S7 Caveolin-1 is synthesized by human HeLa and INT 407 epithelial cells but is not synthesized by human Caco-2 epithelial cells. Cell lysates from HeLa, INT 407, and Caco-2 cells were prepared as described in the ‘Methods’ section. The blots were probed with antibodies reactive against caveolin-1 and actin. The molecular masses of the protein standards are listed in kDa. Click here for file

Additional file 8: Figure S8 Treatment of Caco-2 cells with 1.25, 2.5, 5.0, and 7.5 mM of methyl-β-cyclodextrin (MβCD) reduces *C. jejuni* internalization. The epithelial cells were treated with MβCD for 30 min prior to inoculation with *C. jejuni*, as outlined in the ‘Methods’ section. The control consisted of cells infected with *C. jejuni* in medium containing the vehicle (water). Bars indicate the mean number of internalized bacteria. The asterisks indicate a significant reduction in *C. jejuni* internalization compared to cells infected with *C. jejuni* in the absence of the inhibitor in medium alone, as judged by one-way ANOVA followed by post-hoc Tukey’s analysis (*P* < 0.05). Each error bar represents ± the standard deviation of the mean (SD). Click here for file

Additional file 9: Figure S9 Expression of exogenous caveolin-1 protein does not alter *C. jejuni*-invasion of Caco-2 cells. Panels: A) Binding and internalization of *C. jejuni* in Caco-2 cells. Cells were transfected with nothing (None), caveolin-1 (Cav-1) or an empty vector control (Empty). B) Whole cell lysates of untreated (None), Cav-1 transfected Caco-2 cells, and Caco-2 cells transfected with an empty vector. Caco-2 lysates were probed with an α caveolin-1 antibody. The blot was re-probed with an α tubulin antibody to confirm that equal amounts of protein were loaded into each well. Click here for file

Additional file 10: Figure S10 *C. jejuni* binds to and invades caveolin-1 positive and negative cells with equal efficiency. *C. jejuni* binding and internalization assays were performed with 3T3 mouse embryonic fibroblasts (MEFs) as outlined in 'Methods.' The 3T3 MEF wild-type cell line (3T3 MEF WT, CRL-2752) is Cav-1^+/+^ whereas the 3T3 MEF knockout cell line (3T3 MEF KO, CRL-2753) is Cav-1^-/-^. The numbers of *C. jejuni* bound to and internalized by the 3T3 MEF WT cells versus the 3T3 MEF KO cells were statistically indistinguishable. Click here for file

Additional file 11: Figure S11 Hydroxypropyl-β-cyclodextrin (HPβCD) treatment of HeLa cells disrupts the *C. jejuni*-dependent association of phospho-EGF receptor (pEGFR) with β_1_ integrin. HeLa cells were infected with *C. jejuni* in the presence and absence of 20 mM HPβCD for 45 min. Panels: A) Cell lysates were immunoprecipitated with an EGFR antibody, separated by SDS-PAGE, and blotted for total EGFR (loading control), pEGFR, and β_1_ integrin. HeLa cells were uninfected (Lanes 1 and 3) or *C. jejuni* infected (Lanes 2 and 4) in the absence (Lanes 1 and 2) or presence of HPβCD (Lanes 3 and 4). Also shown are the blots of the IgG isotype control IP probed with antibodies reactive against the EGFR and the β_1_ integrin. B) Quantification of band intensity of pEGFR from three independent experiments. The asterisk indicates *P* < 0.01 by one-way ANOVA followed by post-hoc Dunnet’s analysis. Click here for file

Additional file 12: Figure S12 Methyl-β-cyclodextrin (MβCD) treatment of Caco-2 cells (caveolin-1 negative) prevents EGF receptor (EGFR) activation. Caco-2 cells were infected with *C. jejuni* with and without 5 mM MβCD treatment or uninfected control (Cells only) for 45 min. Panels: A) The cell lysates were immunoprecipitated (IP) with an antibody reactive against the EGFR, separated by SDS-PAGE and blotted for Phospho-EGFR (pEGFR) and total EGFR. IP with an IgG isotype control antibody yielded no reactive bands by immunoblot (not shown). Lanes: 1, Uninfected cells (Cells only); 2, Infected with *C. jejuni* in the absence of MβCD (vehicle only, water); 3, Uninfected cells in the presence of 5 mM MβCD; and 4, Infected with *C. jejuni* in the presence of 5 mM MβCD. B) Quantification by densitometry of the pEGFR bands. The mean ± standard deviation of the pEGFR from three independent blots is indicated in relative optical density. The asterisk indicates *P* ≤ 0.05 as judged by one-way ANOVA followed by post-hoc Dunnet’s analysis. Click here for file
