# Supplementary figures and images for: Invasion of epithelial cells by Campylobacter jejuni is independent of caveolae
Source: Cell Commun Signal. 2013 Dec 23;11:100. doi: 10.1186/1478-811X-11-100 (PMC3880046; doi:10.1186/1478-811X-11-100)

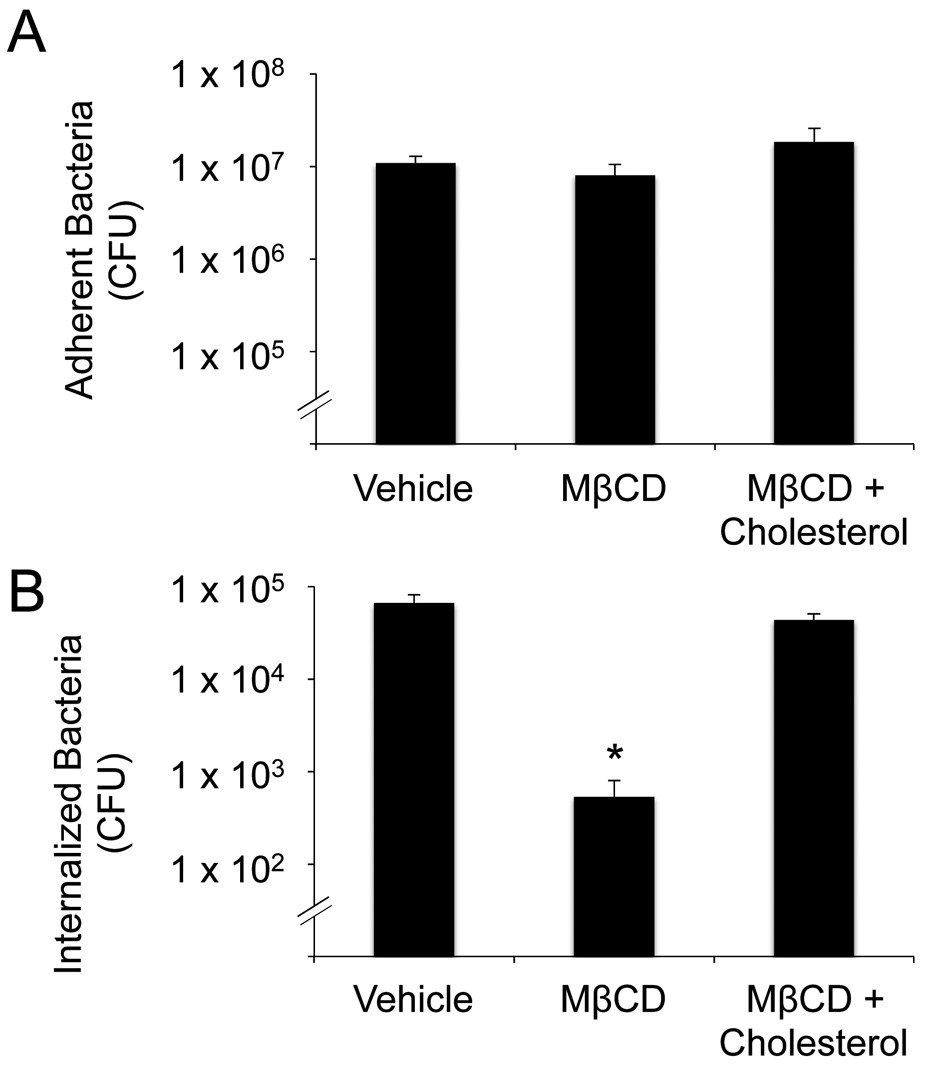

Supplement: Additional file 1: Figure S1 — C. jejuni internalization into HeLa cells treated with methyl-β-cyclodextrin (MβCD) is recovered by cholesterol replenishment. HeLa cells were treated with 5 mM MβCD for 30 min. Membrane cholesterol was then replenished by treatment with cyclodextrin:cholesterol complex for 30 min prior to infection with C. jejuni. Panels: A) Host cell association was unaffected by MβCD treatment and cholesterol replenishment. B) Treatment with 5 mM MβCD significantly reduced C. jejuni internalization. Membrane cholesterol restoration through cyclodextrin:cholesterol complex treatment recovered the invasion phenotype. The asterisk indicates a significant reduction in C. jejuni internalization compared to cells infected with C. jejuni in the absence of the inhibitor (vehicle), as judged by one-way ANOVA followed by post-hoc Dunnets’s analysis (P < 0.05). Each error bar represents ± the standard deviation of the mean (SD). [file 1478-811X-11-100-S1.jpeg]

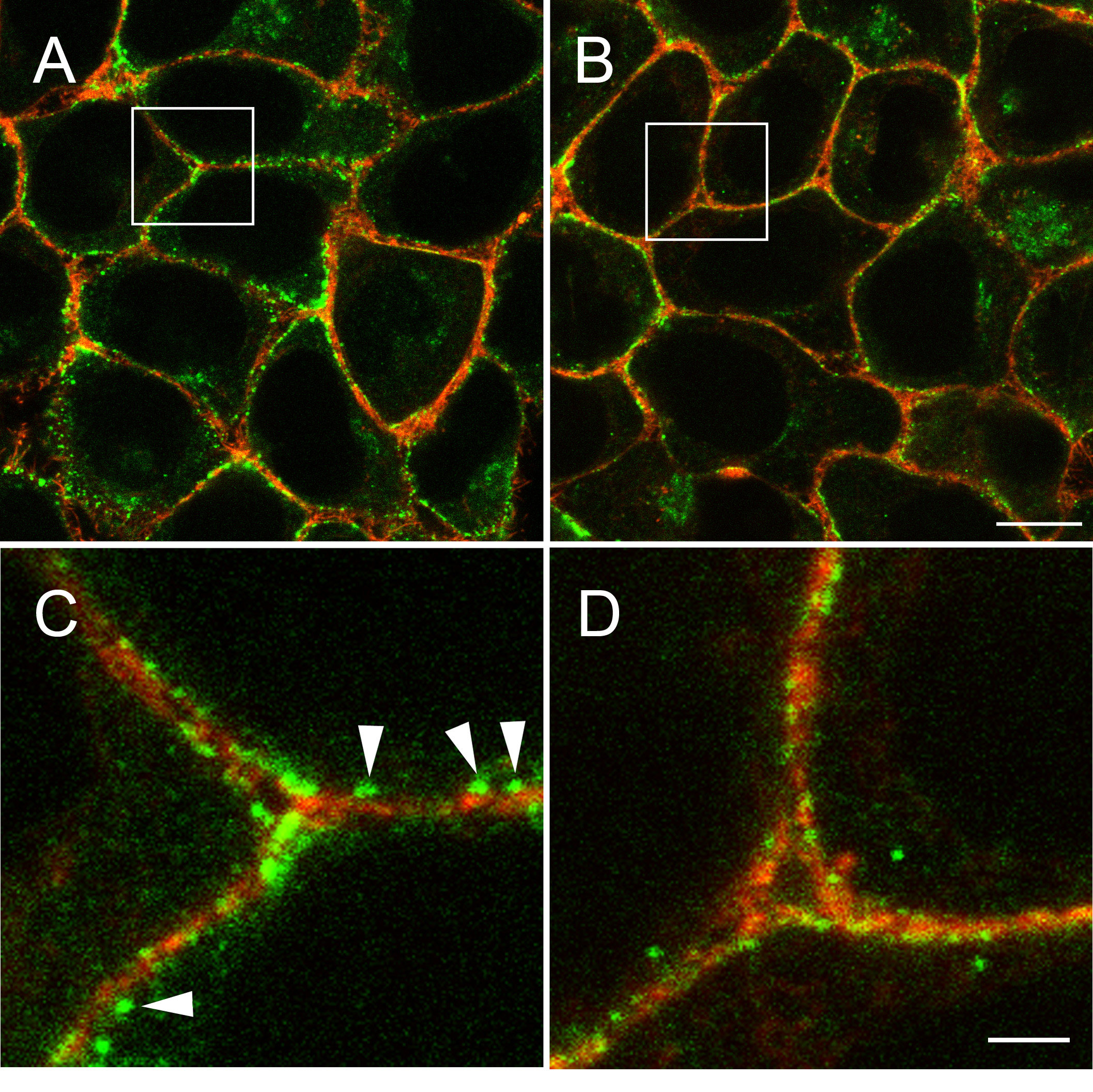

Supplement: Additional file 2: Figure S2 — Effects of methyl-β-cyclodextrin (MβCD) treatment on caveolin-1 localization in HeLa cells. Cells shown were labeled with anti-caveolin primary and fluorescein labeled secondary antibodies (green) and counterstained with rhodamine-labeled phalloidin to visualize filamentous actin (red). Vehicle treated cells (Panels A and C) show abundant caveolin-1 in the vicinity of the cell boundary. Caveolin-1 was localized at or proximal to the cytoplasmic boundary of the actin cytoskeleton underlying the cell plasma membrane (inset in Panel A and arrows in Panel C), suggesting the caveolin-1 may be in the process of recycling. In contrast, MβCD treated cells displayed fewer and smaller sites of caveolin-1 staining (Panels B and D), and appeared more closely associated with actin at the cell boundary (D). Bars are 10 microns (Panels A and B) and 2 microns (Panels C and D). [file 1478-811X-11-100-S2.jpeg]

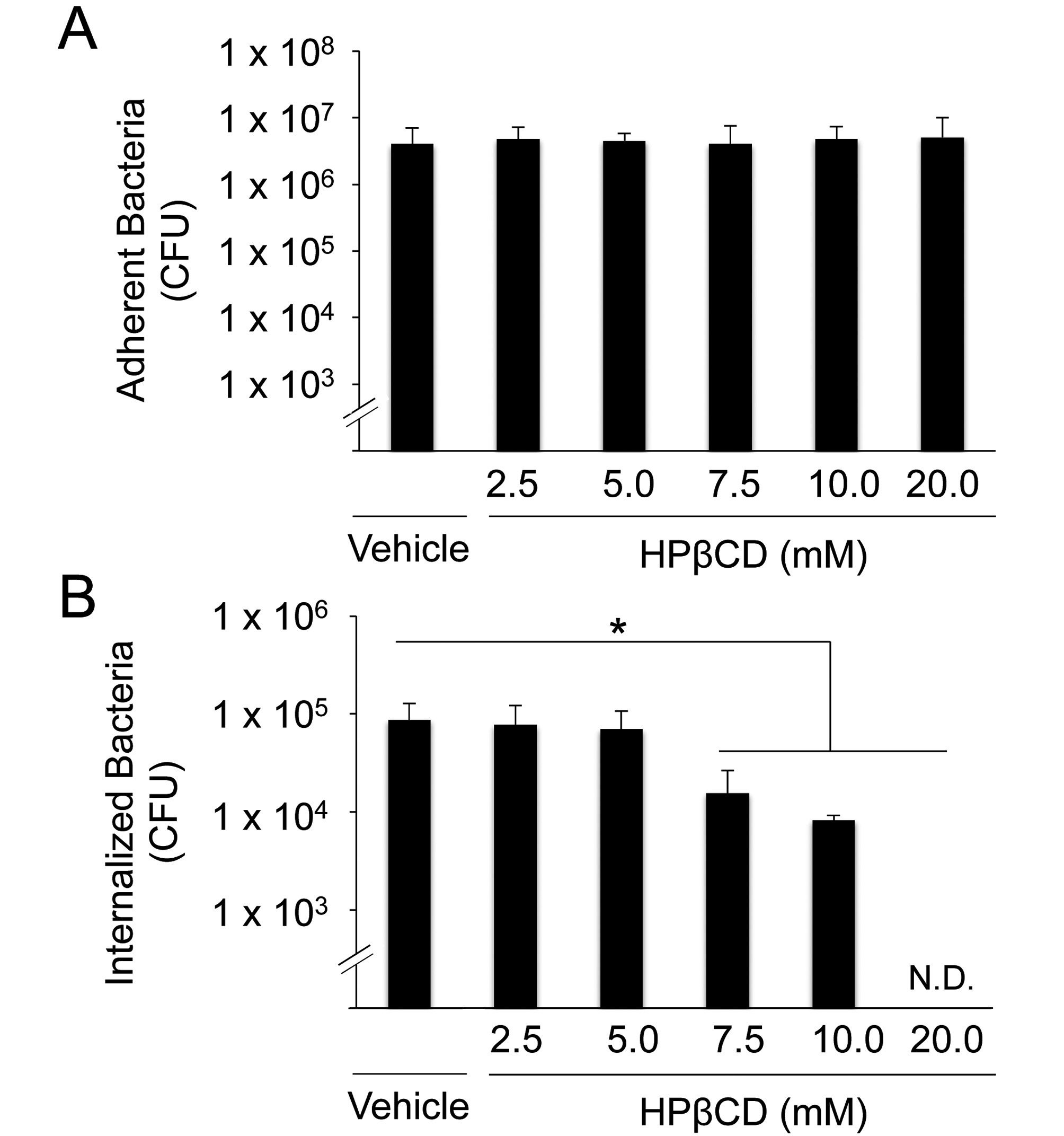

Supplement: Additional file 3: Figure S3 — Treatment of HeLa cells with the cholesterol-depleting compound hydroxypropyl-β-cyclodextrin (HPβCD) reduces C. jejuni internalization. HeLa cells were treated with 2.5, 5.0, 7.5, 10, and 20 mM of HPβCD for 30 min prior to inoculation with C. jejuni. The control consisted of cells infected with C. jejuni in the absence of the inhibitor in medium containing vehicle (i.e., water). Bars indicate the number of adherent (Panel A) and internalized (Panel B) bacteria. The asterisk indicates a significant reduction in C. jejuni internalization compared to cells infected with C. jejuni in the absence of the inhibitor in medium alone, as judged by one-way ANOVA followed by post-hoc Dunnets’s analysis (P < 0.05). Each error bar represents ± the standard deviation of the mean (SD). [file 1478-811X-11-100-S3.jpeg]

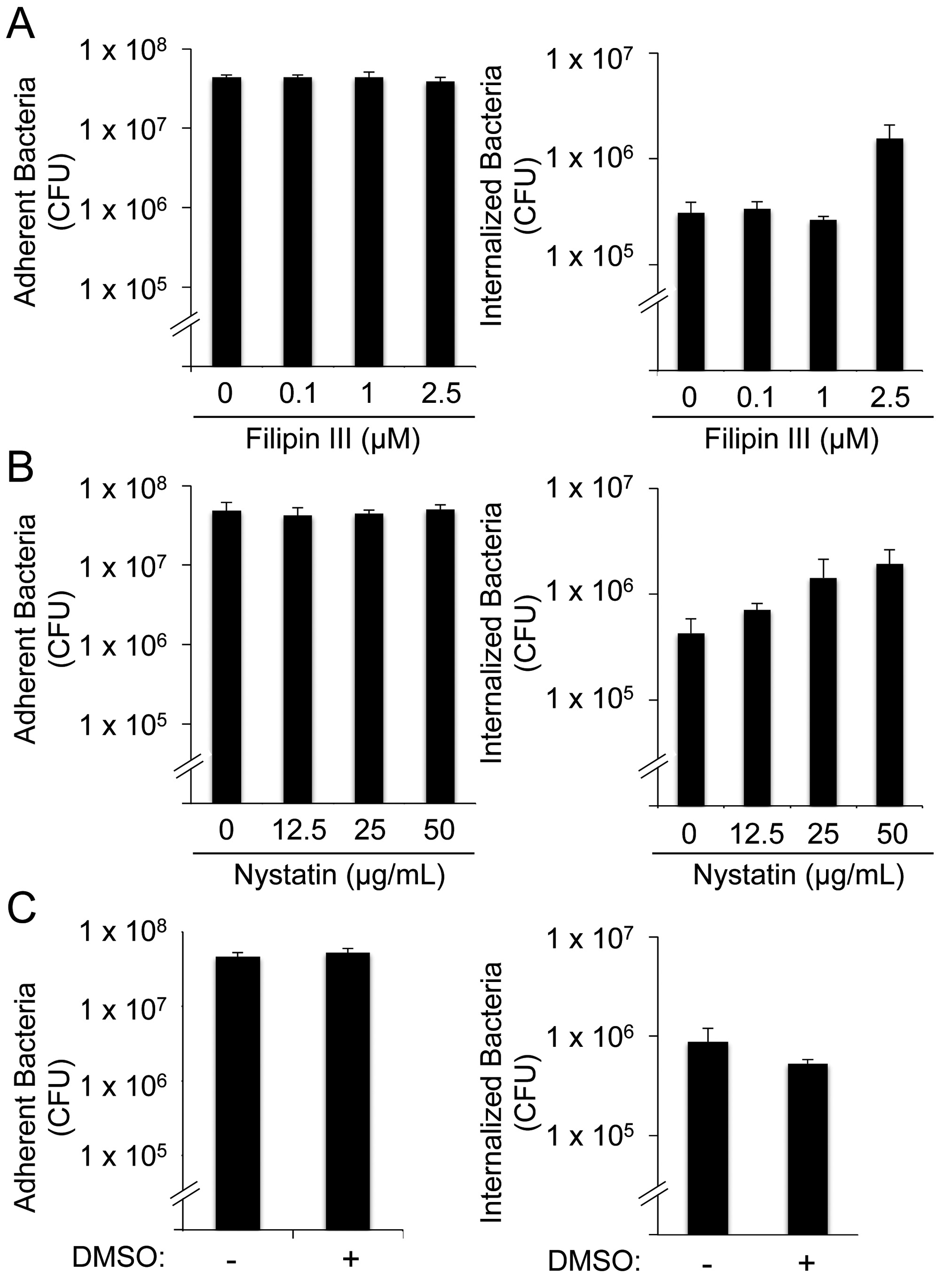

Supplement: Additional file 4: Figure S4 — Treatment of cells with the cholesterol binding agents filipin III or nystatin had no effect on C. jejuni internalization. HeLa cells were treated with a range of concentrations of filipin III (Panel A) and nystatin (Panel B) for 30 min prior to inoculation with C. jejuni. The control consisted of cells infected with C. jejuni in the absence of the inhibitor in medium containing vehicle (i.e., DMSO) (Panel C). Each error bar represents ± the standard deviation of the mean (SD). [file 1478-811X-11-100-S4.jpeg]

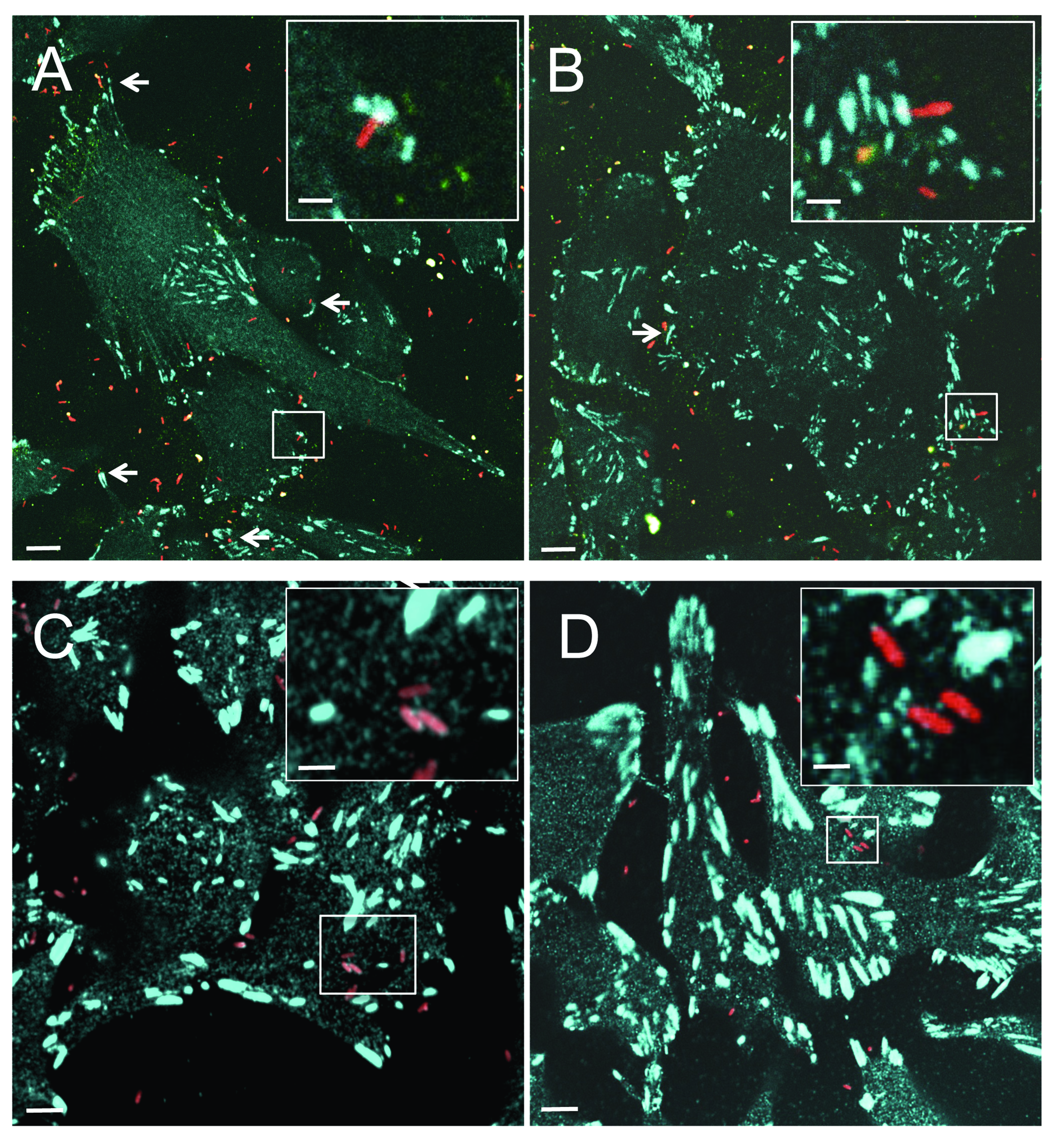

Supplement: Additional file 5: Figure S5 — Methyl-β-cyclodextrin (MβCD) treatment of cells reduces the co-localization of C. jejuni with the focal complex components paxillin and vinculin. HeLa cells were infected with C. jejuni in the absence (Panels A and B) or presence of MβCD (Panels C and D) and examined by confocal microscopy. Paxillin (Panels A and C) and vinculin (Panels B and D) are shown in blue and C. jejuni is shown in red. Also shown is an increased magnification of the image (insert). Sites of co-localization observed in a given field are indicated (arrows). In total, 42.0% of cell-associated C. jejuni were co-localized with paxillin (N = 71 of 169) and 40.3% of cell-associated C. jejuni were co-localized with vinculin (N = 64 of 159 total). Following treatment with MβCD, 25.4% of cell-associated C. jejuni were co-localized with paxillin (N = 33 of 130) and 24.7% of cell-associated C. jejuni were co-localized with vinculin (N = 22 of 89 total). Scale bar is 10 microns for low magnification images and 2 microns for the higher magnification images. [file 1478-811X-11-100-S5.jpeg]

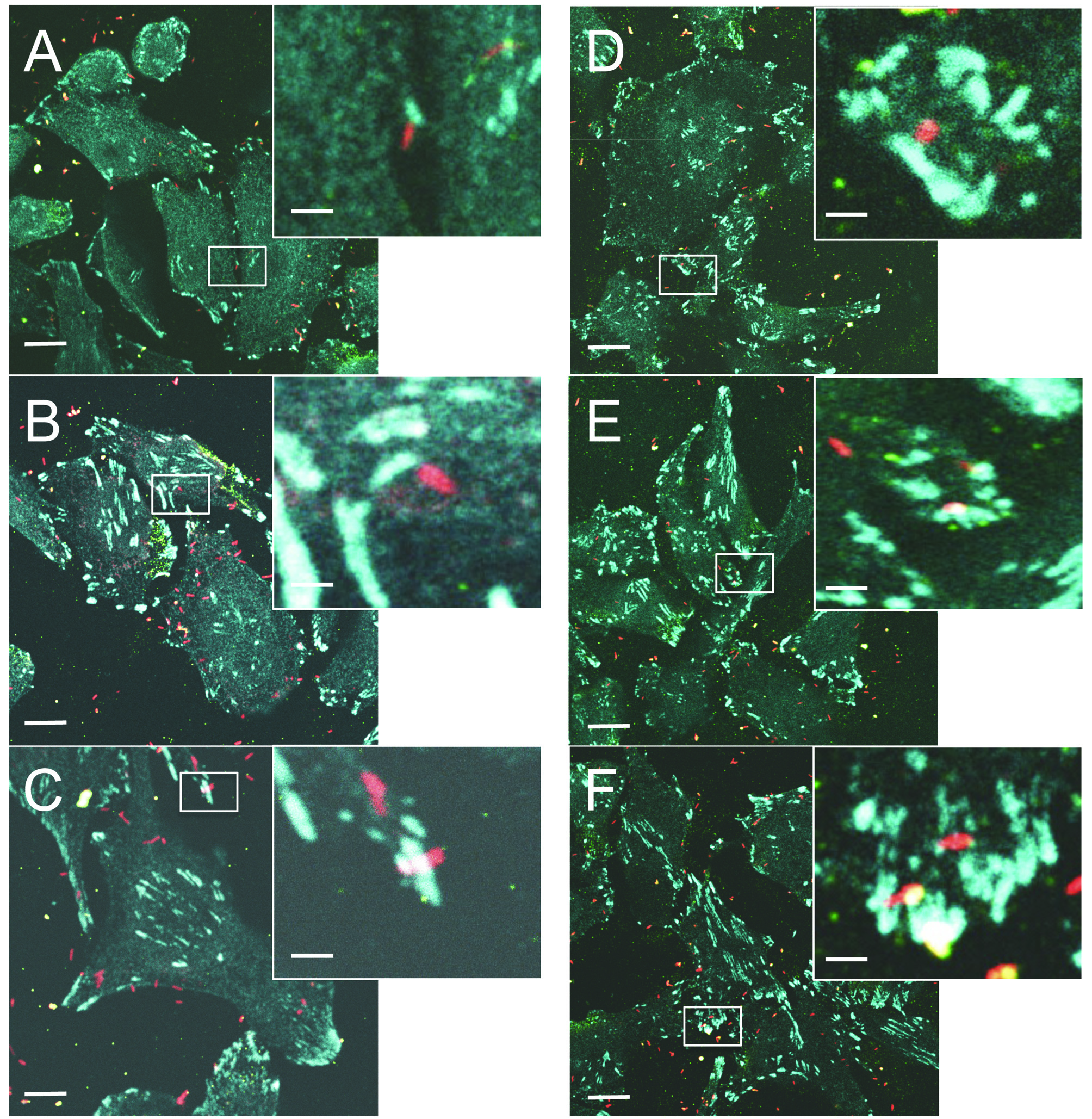

Supplement: Additional file 6: Figure S6 — Additional confocal microscopy images showing C. jejuni associated with paxillin and vinculin. Paxillin (Panels A-C) and vinculin (Panels D-F) are shown in blue and C. jejuni is shown in red. Also shown is an increased magnification of each image (insert). Scale bar is 10 microns for low magnification images and 2 microns for the higher magnification images. [file 1478-811X-11-100-S6.jpeg]

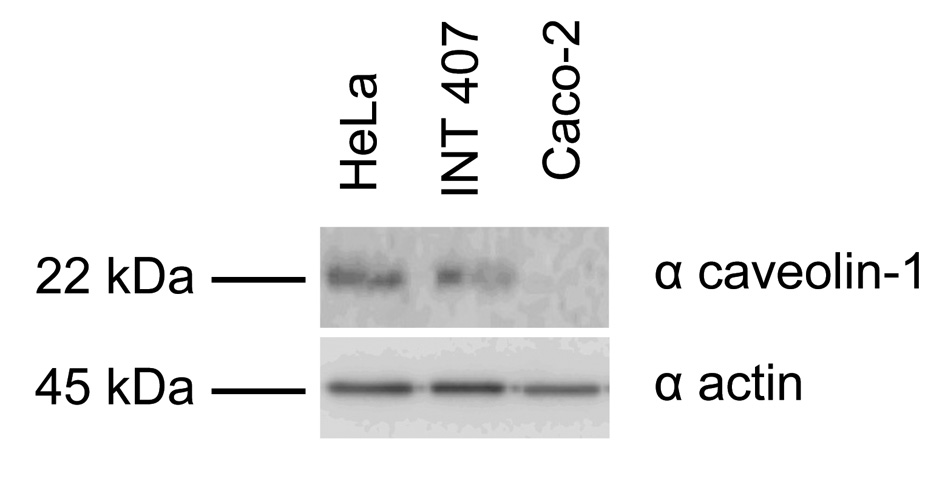

Supplement: Additional file 7: Figure S7 — Caveolin-1 is synthesized by human HeLa and INT 407 epithelial cells but is not synthesized by human Caco-2 epithelial cells. Cell lysates from HeLa, INT 407, and Caco-2 cells were prepared as described in the ‘Methods’ section. The blots were probed with antibodies reactive against caveolin-1 and actin. The molecular masses of the protein standards are listed in kDa. [file 1478-811X-11-100-S7.jpeg]

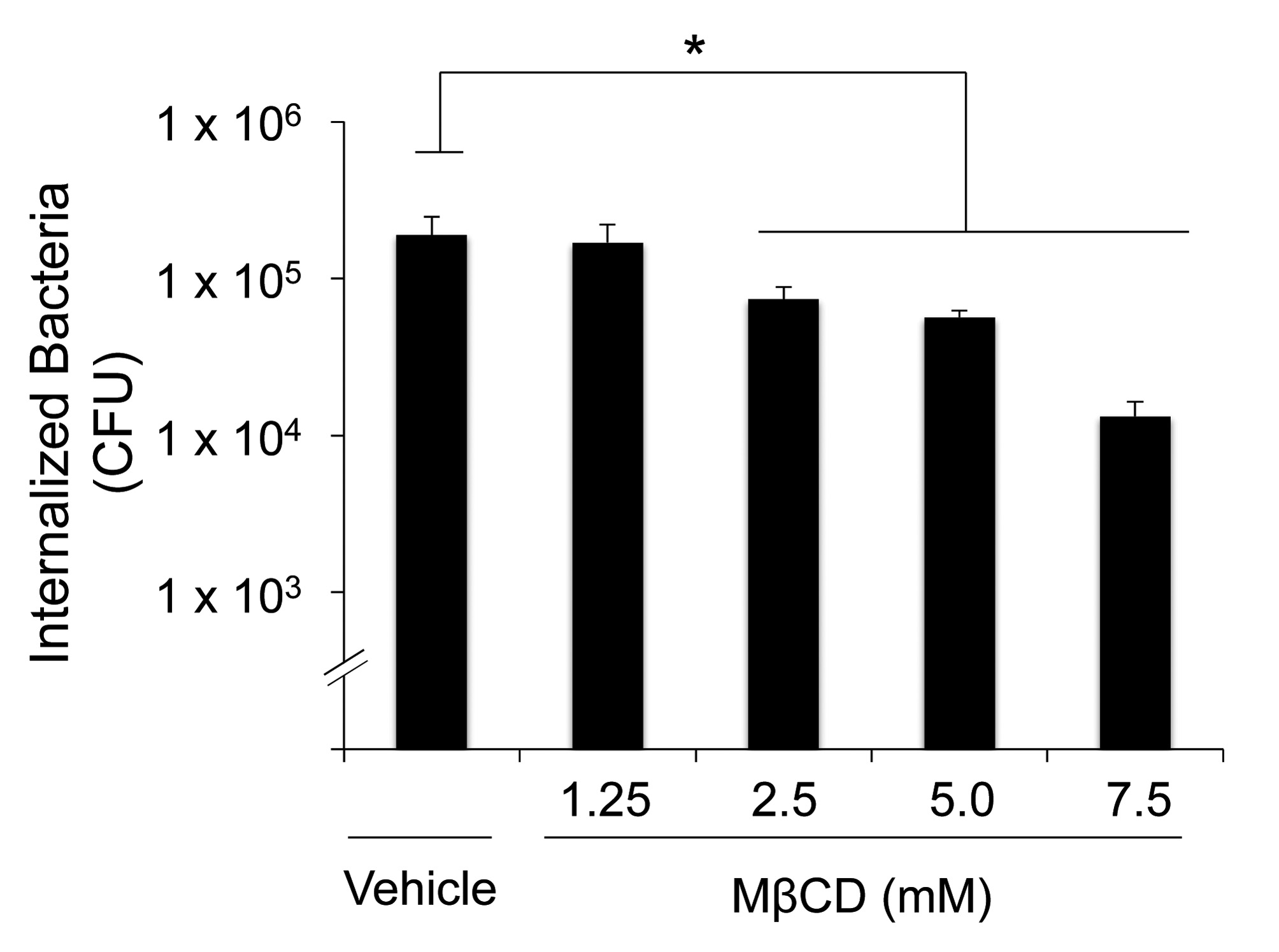

Supplement: Additional file 8: Figure S8 — Treatment of Caco-2 cells with 1.25, 2.5, 5.0, and 7.5 mM of methyl-β-cyclodextrin (MβCD) reduces C. jejuni internalization. The epithelial cells were treated with MβCD for 30 min prior to inoculation with C. jejuni, as outlined in the ‘Methods’ section. The control consisted of cells infected with C. jejuni in medium containing the vehicle (water). Bars indicate the mean number of internalized bacteria. The asterisks indicate a significant reduction in C. jejuni internalization compared to cells infected with C. jejuni in the absence of the inhibitor in medium alone, as judged by one-way ANOVA followed by post-hoc Tukey’s analysis (P < 0.05). Each error bar represents ± the standard deviation of the mean (SD). [file 1478-811X-11-100-S8.jpeg]

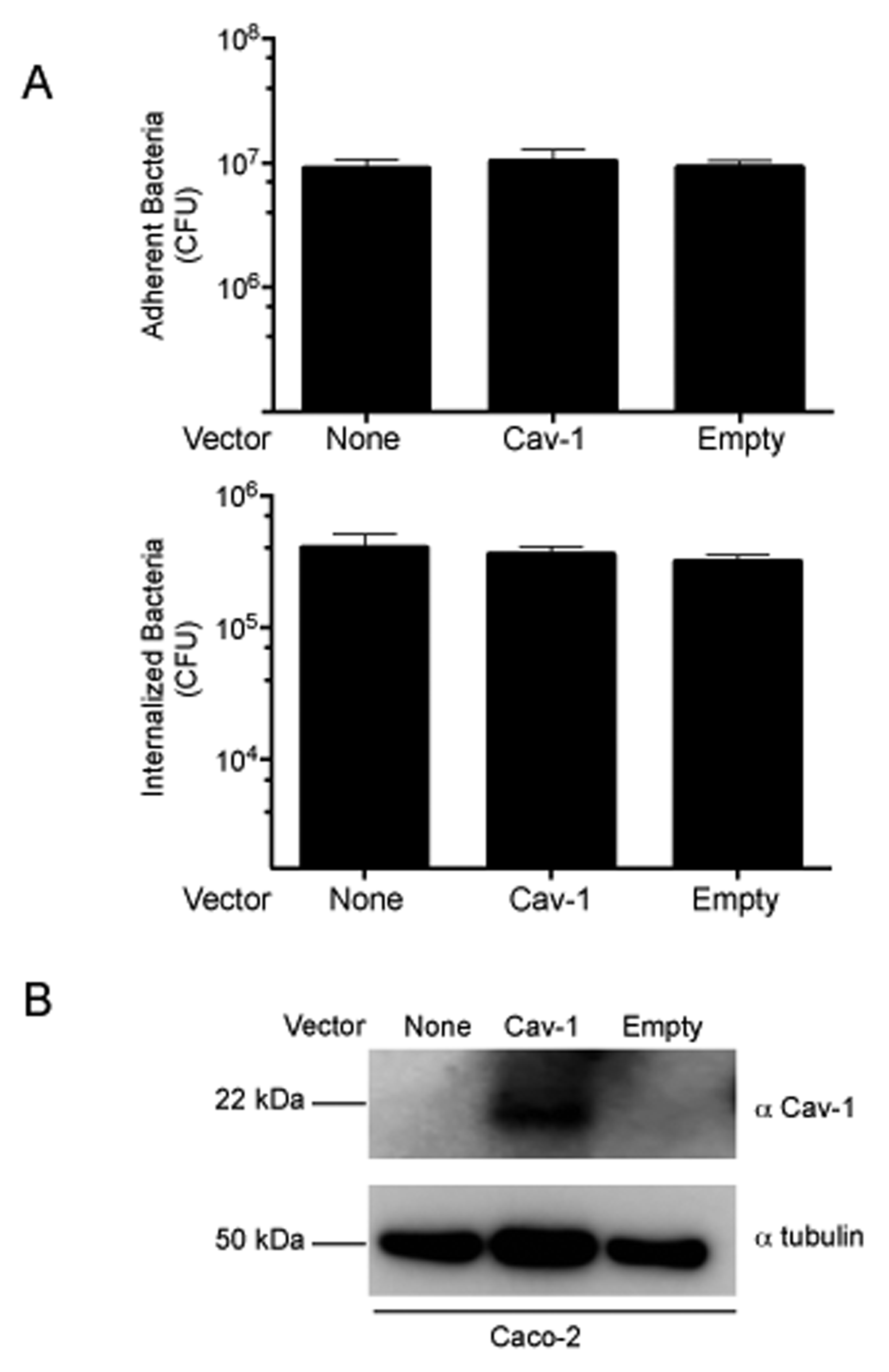

Supplement: Additional file 9: Figure S9 — Expression of exogenous caveolin-1 protein does not alter C. jejuni-invasion of Caco-2 cells. Panels: A) Binding and internalization of C. jejuni in Caco-2 cells. Cells were transfected with nothing (None), caveolin-1 (Cav-1) or an empty vector control (Empty). B) Whole cell lysates of untreated (None), Cav-1 transfected Caco-2 cells, and Caco-2 cells transfected with an empty vector. Caco-2 lysates were probed with an α caveolin-1 antibody. The blot was re-probed with an α tubulin antibody to confirm that equal amounts of protein were loaded into each well. [file 1478-811X-11-100-S9.tiff]

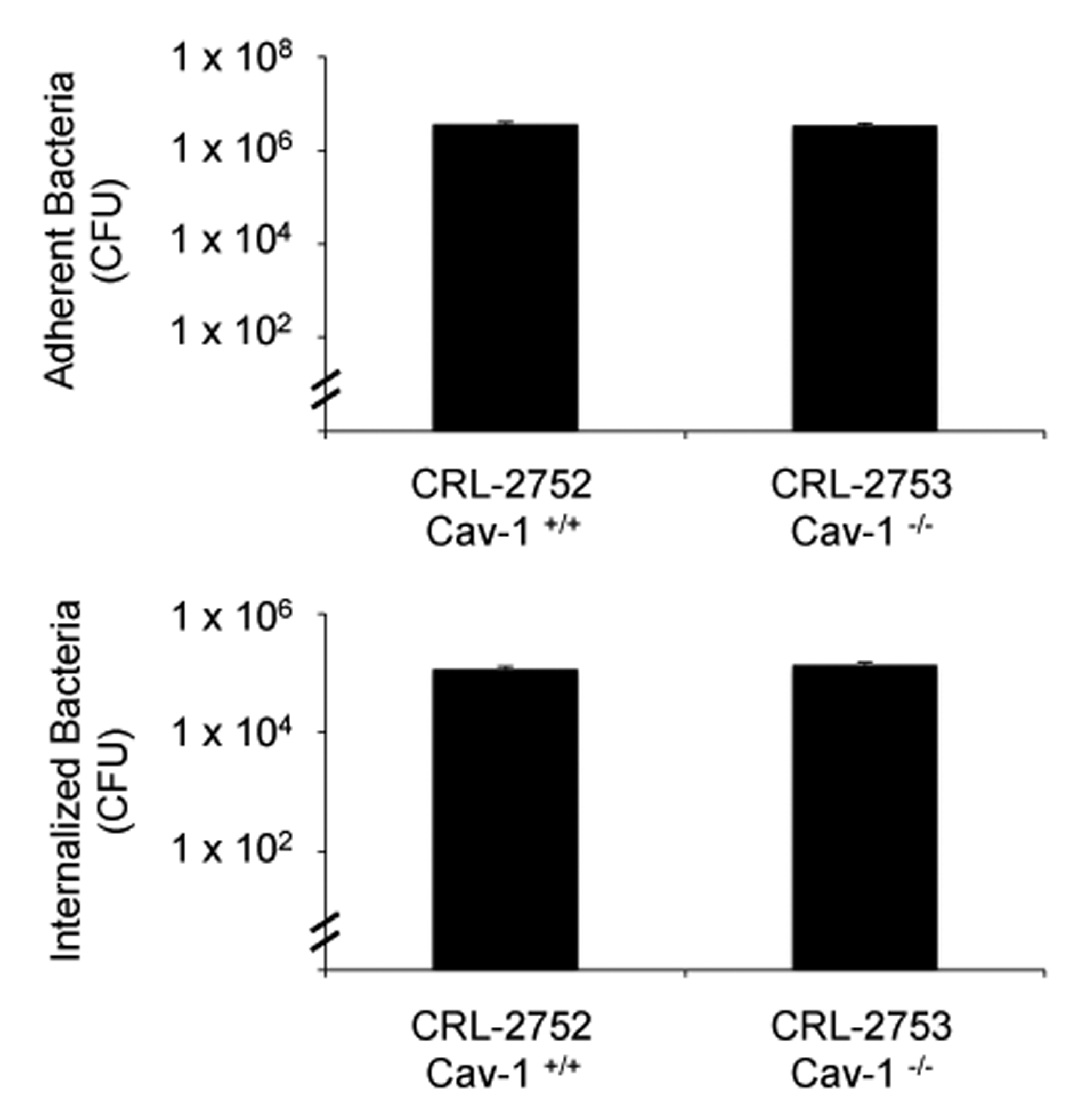

Supplement: Additional file 10: Figure S10 — C. jejuni binds to and invades caveolin-1 positive and negative cells with equal efficiency. C. jejuni binding and internalization assays were performed with 3T3 mouse embryonic fibroblasts (MEFs) as outlined in 'Methods.' The 3T3 MEF wild-type cell line (3T3 MEF WT, CRL-2752) is Cav-1+/+ whereas the 3T3 MEF knockout cell line (3T3 MEF KO, CRL-2753) is Cav-1-/-. The numbers of C. jejuni bound to and internalized by the 3T3 MEF WT cells versus the 3T3 MEF KO cells were statistically indistinguishable. [file 1478-811X-11-100-S10.tiff]

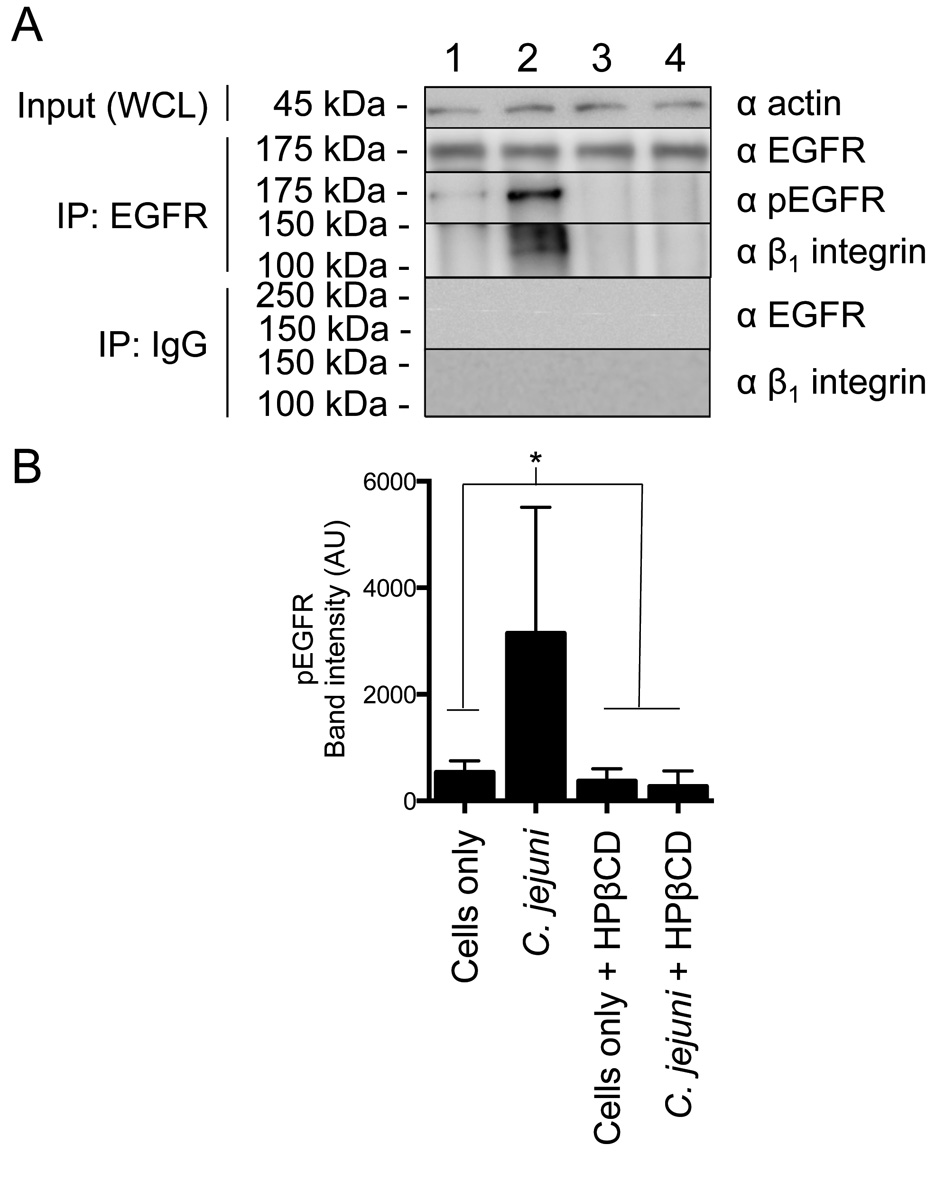

Supplement: Additional file 11: Figure S11 — Hydroxypropyl-β-cyclodextrin (HPβCD) treatment of HeLa cells disrupts the C. jejuni-dependent association of phospho-EGF receptor (pEGFR) with β1 integrin. HeLa cells were infected with C. jejuni in the presence and absence of 20 mM HPβCD for 45 min. Panels: A) Cell lysates were immunoprecipitated with an EGFR antibody, separated by SDS-PAGE, and blotted for total EGFR (loading control), pEGFR, and β1 integrin. HeLa cells were uninfected (Lanes 1 and 3) or C. jejuni infected (Lanes 2 and 4) in the absence (Lanes 1 and 2) or presence of HPβCD (Lanes 3 and 4). Also shown are the blots of the IgG isotype control IP probed with antibodies reactive against the EGFR and the β1 integrin. B) Quantification of band intensity of pEGFR from three independent experiments. The asterisk indicates P < 0.01 by one-way ANOVA followed by post-hoc Dunnet’s analysis. [file 1478-811X-11-100-S11.jpeg]

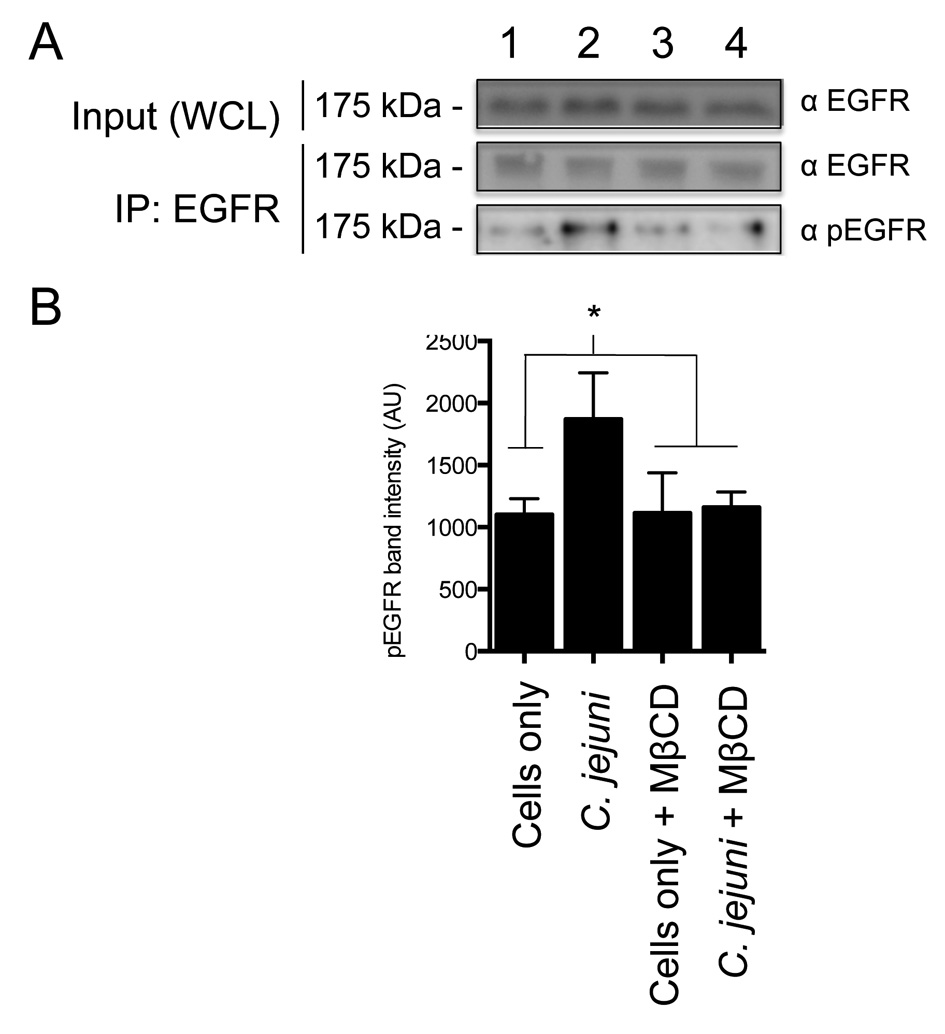

Supplement: Additional file 12: Figure S12 — Methyl-β-cyclodextrin (MβCD) treatment of Caco-2 cells (caveolin-1 negative) prevents EGF receptor (EGFR) activation. Caco-2 cells were infected with C. jejuni with and without 5 mM MβCD treatment or uninfected control (Cells only) for 45 min. Panels: A) The cell lysates were immunoprecipitated (IP) with an antibody reactive against the EGFR, separated by SDS-PAGE and blotted for Phospho-EGFR (pEGFR) and total EGFR. IP with an IgG isotype control antibody yielded no reactive bands by immunoblot (not shown). Lanes: 1, Uninfected cells (Cells only); 2, Infected with C. jejuni in the absence of MβCD (vehicle only, water); 3, Uninfected cells in the presence of 5 mM MβCD; and 4, Infected with C. jejuni in the presence of 5 mM MβCD. B) Quantification by densitometry of the pEGFR bands. The mean ± standard deviation of the pEGFR from three independent blots is indicated in relative optical density. The asterisk indicates P ≤ 0.05 as judged by one-way ANOVA followed by post-hoc Dunnet’s analysis. [file 1478-811X-11-100-S12.jpeg]
